# Advances in modeling periodontal host–microbe interactions: insights from organotypic and organ-on-chip systems

**DOI:** 10.1039/d4lc00871e

**Published:** 2025-01-30

**Authors:** Hardik Makkar, Gopu Sriram

**Affiliations:** a Faculty of Dentistry, National University of Singapore 119085 Singapore sriram@nus.edu.sg; b Center for Innovation & Precision Dentistry, School of Dental Medicine and School of Engineering, University of Pennsylvania Philadelphia PA 19104 USA makkarh@upenn.edu; c Department of Biomedical Engineering, College of Design and Engineering, National University of Singapore 117583 Singapore

## Abstract

Periodontal disease, a chronic inflammatory condition affecting the supporting structures of teeth, is driven by an imbalanced interaction between the periodontal microbiota and the host inflammatory response. Beyond its local impact, periodontal disease is associated with systemic conditions such as diabetes mellitus, cardiovascular disease, and inflammatory bowel disease, emphasizing the importance of understanding its mechanisms. Traditional pre-clinical models, such as monolayer cultures and animal studies, have provided foundational insights but are limited by their physiological relevance and ethical concerns. Recent advancements in tissue engineering and microfluidic technologies have led to the development of three-dimensional (3D) organotypic culture models and organ-on-chip systems that more closely mimic native tissue microenvironments. This review provides an overview of the evolution of methods to study periodontal host–microbe interactions, from simple 2D monolayer cultures to complex 3D organotypic and microfluidic organ-on-chip (OoC) models. We discuss various fabrication strategies, host–microbe co-culture techniques, and methods for evaluating outcomes in these advanced models. Additionally, we highlight insights gained from gut-on-chip platforms and their potential applications in periodontal research and understanding oral-systemic links of periodontal disease. Through a comprehensive overview of current advancements and future directions, this review provides insights on the transformative potential of OoC technology in periodontal research, offering new avenues for studying disease mechanisms and developing therapeutic strategies.

## Abstract 2

This review highlights the advancements in 3D organotypic and organ-on-chip models for studying periodontal host–microbe interactions, offering insights into disease mechanisms and paving the way for novel therapeutic approaches.

## Graphical abstract

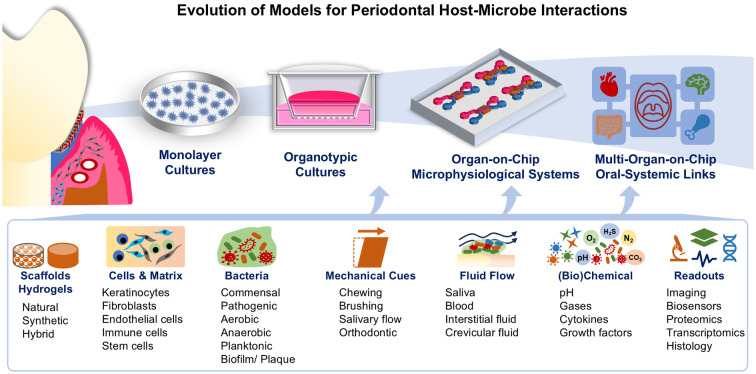

## 1. Introduction

Periodontal disease is a prevalent chronic inflammatory disease that progressively affects the soft and hard tissues that support and anchor the teeth.^1^ It manifests initially as gingivitis, characterized by inflammation of the gingival tissues (gums), and can progress to periodontitis, a more severe condition that leads to the destruction of the periodontal ligament and alveolar bone, resulting in tooth mobility and potential tooth loss.^2^ This disease affects an estimated two-thirds of the adult population, with severe periodontal disease impacting approximately 19%, representing more than one billion individuals worldwide.^3,4^ The World Health Organization has recognized severe periodontal disease as a significant public health concern due to its high prevalence,^5,6^ and its associations with systemic conditions such as diabetes mellitus, cardiovascular disease, and inflammatory bowel disease.^1,7^

The pathogenesis of periodontal disease is primarily driven by microbial dysbiosis and an aberrant host cellular and immune defense response in the gingival and subgingival regions, leading to progressive destruction if left untreated.^1,7,8^ Understanding the complex host–microbe interactions in periodontal disease provides deeper insights into the complex interplay between the host and microbiome, crucial for both periodontal and systemic health. Traditionally, the study of these interactions has relied on monolayer cultures and complex animal models.^9,10^ While the monolayer culture based models have provided foundational insights, they are limited by their reductionistic nature, lack of physiological relevance, and inability to recapitulate the multifactorial interactions between host tissues, microbes, and materials. Alternatively, animal models, primarily using rodents, dogs, and nonhuman primates, have been invaluable for studying periodontitis progression, host–microbe interactions and regenerative strategies. However, their applicability is limited by physiological differences from humans, ethical concerns, high costs, and variations in the diversity of oral microbiome which complicate the direct translatability of findings. These limitations underscore the necessity for more advanced *in vitro* models that can mimic the native periodontal tissue architecture and its dynamic microenvironment.^11^

Advancements in tissue engineering and microfluidic technology have been harnessed to develop three-dimensional (3D) culture systems and organ-on-chip (OoCs) microphysiological platforms.^12^ These innovations offer enhanced physiological relevance by closely emulating the native tissue microenvironments and facilitating the study of complex host–microbe interactions. Recently, these advances have inspired advances in periodontal research and led to the development of 3D tissue-engineered models of the oral mucosa and perfusion-based milli- and microfluidic tissue culture systems to study periodontal host–microbe interactions.^13–15^ Seminal studies on the application of 3D organotypic cultures^16–23^ and microfluidic OoC systems^24–31^ have showcased progressive adaptations towards developing *in vitro* models with increasing complexity to bridge the gap between clinical and preclinical knowledge and better emulate the complexities of the oral microenvironment. These systems incorporate dynamic flow, mechanical cues, and compartmentalized structures, which are crucial for replicating the fluid dynamics, biofilm formation, and nutrient transport observed *in vivo*.

In this review, we aim to provide an overview of the evolution of tissue-engineered cell culture-based *in vitro* models, ranging from monolayer cultures to advanced 3D organotypic and microfluidic OoC systems, to study periodontal host–microbe interactions. We discuss various fabrication strategies, host–microbe co-culture techniques, and readouts used in these advanced models. Furthermore, we explore the advancements in gut-on-chip platforms and the potential insights that could be translated for applications in periodontal research. Despite the distinct differences in functions and microbiota of the gut and periodontium, both tissues share complex host–microbe interactions and critical barrier functions, facilitated by interfaces between aerobic host tissues and anaerobic microbiomes. Insights from gut-on-chip systems, particularly their ability to model dynamic microbial interactions and epithelial responses, along with integrated biosensors for real-time monitoring of biochemical and biophysical outputs, offer valuable strategies for developing more physiologically relevant periodontal models. Through these insights, we aim to provide a comprehensive overview of the current strategies and future directions for *in vitro* models of periodontal health and disease, highlighting the transformative potential of OoC technology in advancing our understanding and treatment of periodontal conditions.

## 2. Periodontal microenvironment: complexities and challenges

**Fig. 1:**
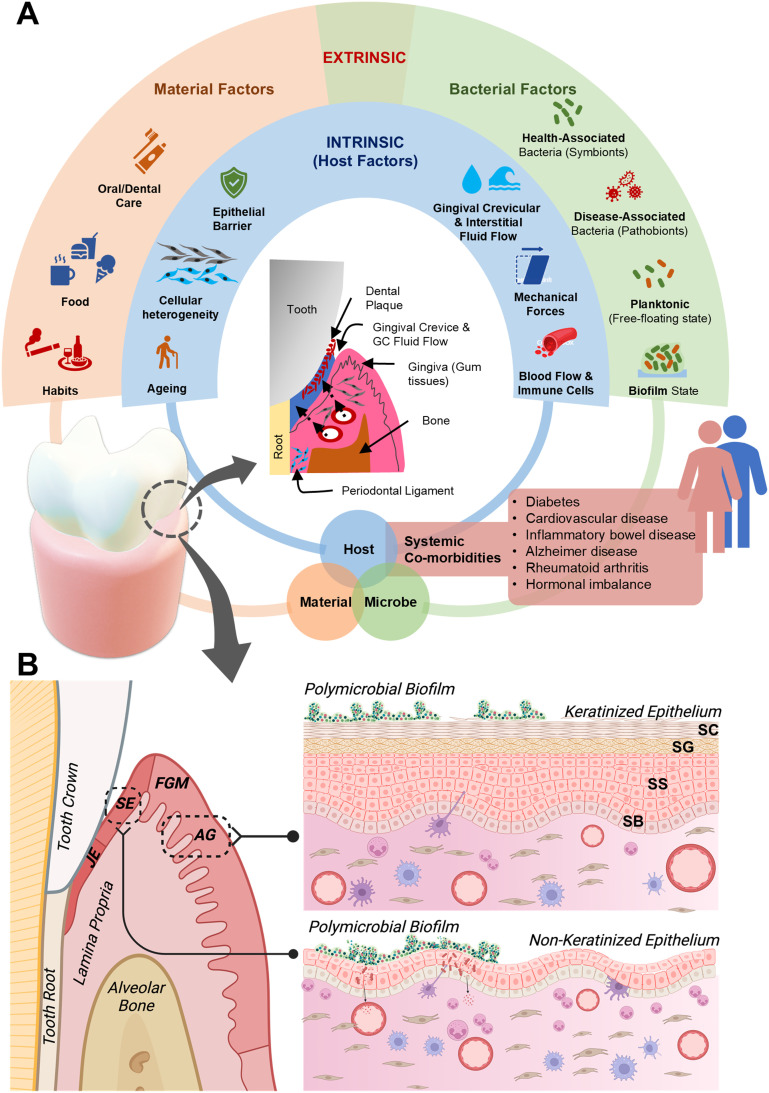
The periodontal microenvironment. (A) The periodontal tissue microenvironment is complex comprising of the gingival soft tissue encircling the tooth and forming a V-shaped sulcus called the gingival crevice. This space harbors the periodontal microbiome which either has a symbiotic or dysbiotic relationship with the host. The host connective tissue fluid known as the gingival crevicular fluid, flows out of this space, bathing the sulcus and rendering host tissue protection. Periodontal health and disease is influenced by intrinsic (host) factors and extrinsic factors. The intrinsic factors comprise of host tissue barrier properties, the influence of cellular heterogeneity in driving immune response, host ageing, innate and adaptive immune system regulating host response to noxious stimuli, and various systemic co-morbidities. The external factors comprise the oral microbiome, its constituents, diversity and state, impact of diet and habits which can directly and indirectly influence host protective responses. (B) Schematic showing the morphological and histological regions of the gingiva that includes attached gingiva (AG), free gingival margin (FGM), sulcular epithelium (SE), and junctional epithelium (JE). Exploded view of AG and SE demonstrating the stratified layers of epithelial cells, namely stratum basale (SB), stratum spinosum (SS), stratum granulosum (SG) and stratum corneum (SC). Below the epithelium is the gingival connective tissue (lamina propria) where gingival fibroblasts are the most abundant cells, responsible for producing the extracellular matrix and collagen fibers. Inflammatory cells, including neutrophils, lymphocytes, and macrophages, play crucial roles in immune defense, tissue remodeling, and responding to periodontal dysbiosis. Endothelial cells line blood vessels and facilitate nutrient exchange and immune cell recruitment. Part B is created with https://Biorendor.com.

The periodontal microenvironment is a dynamic and multifaceted niche comprised of various cellular, microbial, structural, mechanical, and biochemical components that collectively maintain periodontal health. Understanding this environment is crucial for developing effective *in vitro* models to study periodontal disease mechanisms and host–microbe interactions (Fig. 1).

### 2.1. Structural and cellular complexity

The periodontal microenvironment is an intricate system comprising several key tissue types, including the gingiva, periodontal ligament, cementum, and alveolar bone. Each of these tissues contributes distinct cellular populations such as gingival epithelial cells, fibroblasts, osteoblasts, and periodontal ligament cells. The gingival epithelium serves as a primary barrier against microbial invasion,^32^ while the underlying connective tissue provides structural support, and houses vasculature and immune cells crucial for innate defense mechanisms.^33^

Histologically, the gingiva consists of an overlying epithelium and underlying connective tissue. The epithelium, which serves as the primary barrier against the oral microbiome, is divided into oral, sulcular, and junctional epithelium (Fig. 1).^34^ The oral epithelium, including both the attached and free gingiva, features keratinized cells in the masticatory mucosa, providing mechanical strength. In contrast, the sulcular and junctional epithelium are non-keratinized, with the junctional epithelium closely adapted to the tooth surface, contributing to sealing and attachment functions.^2,34^ Further, the gingival sulcus, a shallow crevice between the tooth and free gingiva, is a critical interface between the host and the microbial community residing on the tooth surface. This sulcus harbors the subgingival plaque and is bathed in gingival crevicular fluid (GCF), a serum-like fluid that flows out of the gingival sulcus (Fig. 1).^35^

The gingival connective tissue, or lamina propria, is crucial for epithelial homeostasis, providing structural support and regulating immune functions. This tissue is highly heterogeneous, composed of gingival fibroblasts, periodontal ligament fibroblasts, endothelial cells (blood vessels), and immune cells (Fig. 1).^33^ Gingival fibroblasts, as the predominant cell type, play a critical role in the production and remodeling of the extracellular matrix (ECM), and modulating epithelial morphogenesis and immune responses. The ECM composed of collagen, elastin, glycoproteins, and proteoglycans, provides structural support and mediates biochemical signaling,^36,37^ cell–matrix interactions,^38^ and response to microbial presence.^39^ Collectively, these diverse cellular and matrix components of the gingival connective tissue work to maintain host homeostasis and serves as a connecting link between the oral and the systemic environment.^33,40,41^

### 2.2. Biochemical and mechanical interactions

A critical feature of the periodontal niche is the constant flow of GCF^35^ and a diverse oral microbiome^42^ residing within the gingival crevice. The flow of GCF into the gingival sulcus is vital for protecting periodontal tissues from bacterial invasion, aiding in the clearance of subgingival plaque.^35^ Further, the GCF flow enables the exchange of biochemical signals, including cytokines and growth factors, and immune cells which modulate immune responses and tissue homeostasis.^43–45^ Additionally, the periodontal region experiences various mechanical forces, including those from chewing and oral hygiene practices, which influence cellular responses, tissue remodeling, and GCF flow (Fig. 1).^46^ These forces combined with the biochemical milieu of GCF, create a dynamic environment that significantly impacts periodontal health and disease progression. Replicating these complex biochemical and mechanical cues *in vitro* is challenging yet essential for developing accurate models of periodontal disease.

### 2.3. Microbial ecology and host–microbe interactions

The periodontal environment hosts a highly diverse microbial community, forming complex biofilms on tooth surfaces and within the gingival crevice. This biofilm state provides microorganisms with protection against both mechanical disruption and host immune responses. More than 700 bacterial species form complex communities known as oral biofilms, which are structured into supragingival and subgingival biofilms based on their location relative to the gum line.^2,8,41,47^ Supragingival biofilms are located on and above the gum line, while subgingival biofilms reside below the gum line, within the gingival crevice or periodontal pocket (Fig. 1).

The composition of these biofilms varies significantly across anatomical sites, influenced by the specific microenvironmental conditions that dictate the microbial inhabitants best suited for each niche. Supragingival biofilms predominantly contain facultative anaerobes (symbionts), largely from the *Streptococcus* genus, whose metabolic activities and properties are influenced by the host's diet and saliva. In the gingival crevicular area, biofilms demonstrate greater microbial diversity (symbiotic and pathogenic bacteria), comprising both Gram-positive and Gram-negative facultative anaerobes, as well as Gram-negative obligate anaerobes, which vary depending on the depth of the crevice, health, and disease status.^7,8,48^ The balance between symbiotic and pathogenic microbial populations is crucial for maintaining periodontal health.

### 2.4. Challenges in recapitulating the periodontal microenvironment

**Fig. 2:**
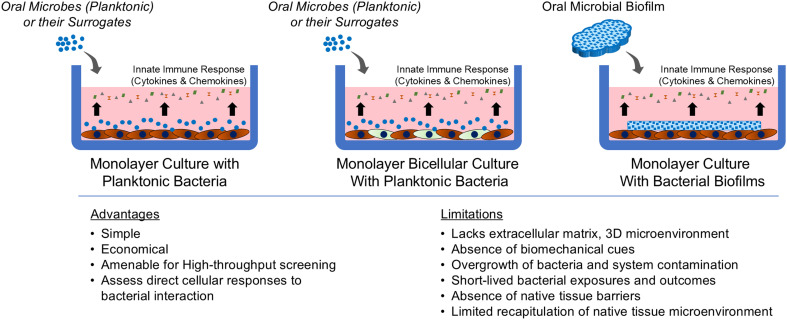
Contribution of monolayer cultures to study periodontal host–microbe interaction. Schematic showing the different techniques employed to expose microbes to cells grown in two-dimension in a culture apparatus. This includes exposing bacteria or their surrogates in planktonic state to single cell type monolayer, bicellular cultures (two different types of adherent cells), and exposing cells to bacterial biofilms grown on substrates. The key advantages and limitations are listed below the schematic.

Replicating the intricate periodontal microenvironment *in vitro* using traditional culture systems presents several challenges. Historically, monolayer culture-based models have served as foundational tools for studying periodontal host–microbe interactions, primarily by allowing direct exposure of host cells to planktonic microbes (Fig. 2). These models typically involve the application of a controlled concentration of microbes to a confluent layer of host cells, such as keratinocytes,^49–51^ gingival fibroblasts,^52–54^ periodontal ligament fibroblasts,^55–57^ and endothelial cells,^58–60^ using the principle of multiplicity of infection (MOI), and record the dose- and time-dependent interactions.^61^ While these models offer simplicity, cost-effectiveness, and suitability for high-throughput screening, they are inherently limited by their reductionistic nature and lack of physiological relevance.^10,62^ Additionally, these models are limited by the short duration of microbial exposure (usually 4–24 hours)^63–65^ due to the toxic by-products from bacterial metabolism that can damage host cells.^66,67^ Although these models provide valuable insights into cellular responses to microbial adhesion and invasion,^66,68^ and associated innate immune response responses,^69–71^ they fail to capture the complex, 3D architecture and cellular heterogeneity of native gingival tissues. Further, the absence of an ECM and a dynamic microenvironment leads to an incomplete representation of the multifactorial interactions occurring *in vivo*. Alternatively, animal models are advantageous in that they can accurately simulate the comprehensive progression of periodontitis, from the initial stages of microbial colonization to advanced tissue destruction (readers can refer to excellent reviews elsewhere^72,73^). These features provide valuable insights into the complex host–microbe interactions and disease dynamics including microbial diversity of subgingival plaque reflecting different states of health, gingivitis, and periodontitis. However, the direct applicability of findings from animal models to human conditions is limited by significant physiological and anatomical differences.^73^ For instance, discrepancies in plaque biofilm composition, saliva properties, and dental anatomy, such as the continuously growing incisors in rodents impacts translatability. Additionally, the intricate biology of these models also poses challenges in isolating and analyzing specific factors systematically. Beyond scientific considerations, ethical issues, and high costs further underscore the need for developing alternative models that offer more human-relevant insights.

To address these limitations inherent in monolayer cultures and animal models, and to more accurately mimic the periodontal microenvironment, advancements in tissue engineering have facilitated the development of 3D organotypic cultures.^74–77^ These models overcome the constraints of monolayer cultures by incorporating multiple cell types within a structured ECM, enabling the study of complex cell–cell and cell–matrix interactions. However, a significant challenge is the need for the integration of biomechanical cues and physiological fluid dynamics, both of which are crucial for replicating the native environment of periodontal tissues. Further, maintaining complex host–microbiome interactions *in vitro* demands the integration of co-culture strategies that reflect the native oxygen gradients and nutrient availability. Moreover, recreating the anaerobic conditions necessary for the co-culture of periodontal pathogens alongside aerobic host cells presents technical challenges in the current organotypic culture systems.

## 3. Periodontal host–microbe interactions: insights from 3D organotypic cultures

3D organotypic tissue equivalents provide a more physiologically relevant platform by closely replicating the complex architecture of native gingival tissues. These models incorporate multiple cell types within a structured ECM, enabling the study of complex cell–cell and cell–matrix interactions crucial for understanding periodontal health and disease. A key advantage of 3D organotypic cultures is their ability to simulate the multi-layered cellular organization found in gingival tissues, including stratified squamous epithelium and underlying connective tissues. Further, the air–liquid interface culture employed in these models allows the exposure of tissue constructs to both planktonic and biofilm bacteria including single and multi-species communities. The application of organotypic cultures that include oral mucosal equivalents, gingival equivalents, and connective tissue equivalents to study periodontal host–microbe interactions are summarized in Tables 1–3.

**Table 1:** Summary of studies related to the use of reconstructed human oral mucosal equivalent for oral host–microbe interaction studies

Author	Oral microbes	Organotypic model	Readouts	Key results
Andrian *et al.* 2004 (ref. 78)	*P. gingivalis*	Primary epithelial and fibroblasts cells in collagen hydrogel	TEM, ELISA	• Higher invasion of nonmutant form into lamina propria
				• Increased secretion of cytokines following microbial exposure
Kimball *et al.* 2006 (ref. 79)	*P. gingivalis*; *S. gordonii*, *Fusobacterium nucleatum*	EpiOral™ (MatTek)	H&E, IHC, RT-PCR	• Epithelial response in the form of increase of hBD2 expression after microbial exposure
Andrian *et al.* 2007 (ref. 80)	*P. gingivalis*	Primary epithelial and fibroblasts cells in collagen hydrogel	RT-PCR, ELISA	• Increased expression of TIMP-2, MMP-2 and MMP-9 by tissue equivalent following microbial exposure
Gursoy *et al.* 2010 (ref. 81)	*F. nucleatum*	HaCaT epithelial cells grown on a fibroblast-populated collagen matrix	H&E, Ki-67, LDH release	• Bacterial invasion of the collagen matrix
				• Biofilms exhibited greater cytotoxicity and invasiveness compared to planktonic bacteria
Pollanen *et al.* 2012 (ref. 82)	*F. nucleatum*	HaCaT cells seeded on collagen gel incorporaed with fibroblasts	IHC	• Epithelial migration and altered epithelial proliferation pattern
Wayakanon *et al.* 2013 (ref. 50)	*P. gingivalis*	Normal oral keratinocytes or TR146 cell on collagen enmeshed fibroblasts	Bacteria count, IHC	• Decreased intracellular levels of *P. gingivalis* observed with polymersome-encapsulated metronidazole or doxycycline treatment
Pinnock *et al.* 2014 (ref. 65)	*P. gingivalis*	Normal oral keratinocytes or H357 cell line on collagen containing NOFs	Antibiotic protection assay, IF, IHC, chemokine array	• Enhanced intracellular survival of *P. gingivalis* in mucosal models relative to monolayer cultures
De Ryck *et al.* 2014 (ref. 83)	Microbiota derived from a swab of the inner cheek	TR146, HaCaT, or normal keratinocytes grown on collagen layer containing NIH-3 T3 fibroblasts	Scratch assay, live/dead staining, metabolites (lactate, acetate), pH, LDH release, Western blot, H&E	• Impaired healing observed in the presence of microbiota
Bugueno *et al.* 2018 (ref. 84)	*P. gingivalis*	3D microtissue of OKF6/TERT-2 cell line on 3D spheroid of normal oral fibroblasts	Antibiotic protection assay, RT-PCR, IF, SEM, TEM	• Microbial exposure led to invasion of the fibroblastic spheroid core and elevated apoptosis
Shang *et al.* 2019 (ref. 16)	Commensal, gingivitis, or cariogenic biofilms from human saliva	Oral keratinocytes (KC-TERT, OKG4/bmi1/TERT) on collagen-embedded fibroblast (Fib-TERT)	FISH, H&E, RT-PCR, Western blotting	• Commensal biofilm induced upregulation of genes associated with TLR signaling
				• Stable epithelial morphology after biofilm exposure
Ingendoh-Tsakmakidis *et al.* 2019 (ref. 85)	Biofilms of *S. oralis* on polyethersulfone membrane, *A. actinomycetemcomitans* (JP2 strain) on coverslip	RhOME (OKF6/TERT-2 seeded on titanium disks coated with gingival fibroblast-incorporated collagen matrix)	Microarray, ELISA, IHC	• *S. oralis* triggered a protective stress response
				• *A. actinomycetemcomitans* caused downregulation of genes related to the inflammatory response

**Table 2:** Summary of studies related to the use of reconstructed human gingival equivalent (RhGE) for oral host–microbe interaction studies

Author	Oral microbes	Organotypic model	Readouts	Key results
Belibasakis *et al.* 2013 (ref. 20)	*P. gingivalis*; *C. rectus*; *F. nucleatum*	EpiGing™ (MatTek)	RT-PCR, LDH release, ELISA	• Increased gene expression and IL-8 secretion after 3 h of red complex biofilms exposure
	*P. intermedia*; *T. forsythia*; *T. denticola*			
	*V. dispar*; *A. oris*; *S. anginosus*; *S. oralis*			
Thurnheer *et al.* 2014 (ref. 86)	*P. gingivalis*; *S. oralis*; *S. anginosus*	EpiGing™ (MatTek)	IF, confocal microscopy, SEM, histological staining	• Colonization of tissue equivalent by “red-complex” species
	*A. oris*; *F. nucleatum*; *V. dispar*			
	*C. rectus*; *P. intermedia*			
	*T. forsythia*; *T. denticola*			
Bao *et al.* 2015 (ref. 63)	*P. gingivalis*; *P. intermedia*	RhGE (bioreactor system with immortalized epithelial cells, fibroblasts, and a monocytic cell line integrated into a 3D collagen scaffold)	Proteomics, LC-MS/MS analysis, gene ontology analysis	• Detected 896 proteins in the supernatant and 3363 in the biofilm lysate
	*A. actinomycetemcomitans* JP2			
	*C. rectus*; *V. dispar*; *F. nucleatum*			
	*S. oralis*; *T. denticola*; *A. oris*			
	*S. anginosus*; *T. forsythia*			
Bao *et al.* 2015 (ref. 23)	*P. gingivalis*; *P. intermedia*	RhGE (bioreactor with 3D collagen scaffold incorporating immortalized epithelial cells (HGEK-16), fibroblasts (FB-16), and a monocytic cell line)	RT-PCR, ELISA, Masson's trichrome staining, SEM	• Decreased growth of *Campylobacter rectus*, *Actinomyces oris*, *S. anginosus*, *Veillonella dispar*, and *P. gingivalis* with OMM
	*A. actinomycetemcomitans* JP2			• Elevated cytokine levels in culture supernatants post biofilm exposure
	*C. rectus*; *V. dispar*; *F. nucleatum*			
	*S. oralis*; *T. denticola*; *A. oris*			
	*S. anginosus*; *T. forsythia*			
Brown *et al.* 2019 (ref. 87)	*P. gingivalis*; *S. mitis*; *S. intermedius*	RhGE (Episkin, Skinethic, Lyon, France)	H&E, LDH assay, RT-PCR, ELISA	• HGE maintained high viability across all multispecies biofilms
	*S. oralis*; *F. nucleatum*; *A. naeslundi*			• Immune cells exhibited varied inflammatory responses when cultured with epithelium and exposed to ‘gingivitis-associated’ biofilm
	*P. intermedia*; *A. actinomycetemcomitans*			
Dabija-Wolter *et al.* 2012 (ref. 19)	*F. nucleatum*	RhGE (primary gingival keratinocytes and fibroblasts)	Confocal microscopy, IHC, RT-PCR	• *F. nucleatum* infiltrated the gingival epithelium without affecting cell viability
Buskermolen *et al.* 2018 (ref. 21)	Commensal, gingivitis, and cariogenic biofilms	RhGE (collagen hydrogel with immortalized human keratinocytes (KC-TERT) and fibroblasts (Fib-TERT))	IHC, FISH, FRET, ELISA	• Elevated elafin expression
				• Gingiva epithelium secreted antimicrobial and inflammatory cytokines
Shang *et al.* 2018 (ref. 17)	Microbial sampling from healthy human saliva consisting of commensal oral microbes	RhGE (immortalized human keratinocyte (KC-TERT) and fibroblast (fib-TERT)-populated hydrogel)	ELISA, RT-PCR, CFU count, H&E, FISH	• Biofilm-exposed RHG showed greater epithelial thickness, stratification, keratinocyte proliferation, and antimicrobial protein production
Shang *et al.* 2019 (ref. 16)	Commensal, gingivitis, or cariogenic biofilms	RhGE (collagen embedded with fibroblasts (fib-TERT) with keratinocytes (KC-TERT, OKG4/bmi1/TERT) layered on top)	FISH, H&E, RT-PCR, western blotting	• Commensal biofilm induced upregulation of genes related to TLR signaling
				• Stable RHG morphology after biofilm exposure
Beklen *et al.* 2019 (ref. 88)	*A. actinomycetemcomitans*	RhGE (immortalized human gingival keratinocyte cells seeded on fibroblast–collagen matrix)	IHC, TEM	• Thick necrotic layer and decreased keratin expression in epithelium following infection
Lin Shang *et al.*^89^	*S. mitis*	RhGE (immortalized human gingival keratinocyte cells seeded on fibroblast–collagen matrix)	FISH, H&E, ELISA, RT-PCR, western blotting	• Weak innate immune response by tissue equivalent with and without the presence of nickel and titanium
				• Immune responses compared with skin
Xiaolan Li *et al.* 2021 (ref. 90)	Mixed species biofilms	RhGE (immortalized human keratinocyte (KC-TERT) and fibroblast (Fib-TERT)-populated hydrogel)	16sDNA sequencing	RhGE supported highly viable and diverse biofilms
Zhang Y *et al.* 2022 (ref. 91)	*A. actinomycetemcomitans*	RhGE (immortalized human keratinocyte (KC-TERT) and fibroblast (fib-TERT)-populated hydrogel)	FISH, H&E, ELISA	• Increased secretion of pro inflammatory cytokines and antimicrobial peptides after exposure with *A. actinomycetemcomitans*
	*S. Gordoni*			
				• *S. Gordoni* exposure led to maximum elafin secretion
Golda A. *et al.* (2024)^92^	*P. gingivalis*	RhGE immortalized human keratinocyte	RT-PCR, H&E, IHC	• Intraepithelial invasion by *P. gingivalis*
		Immortalized human fibroblasts		• Elimination of pathogen on tissue equivalent by specific antimicrobial

**Table 3:** Summary of studies related to the use of connective tissue equivalents (CTEs) for oral host–microbe interaction studies

Author	Oral microbes	Organotypic model	Readouts	Key results
Hillman *et al.* 1999 (ref. 93)	NA	Primary gingival fibroblasts cultured on Fibra-Cel carriers	Light microscopy, IF, SEM, confocal microscopy, TEM	• 3D culture conditions led to elongated and stellate fibroblast morphology
				• Expression of collagen type I, III, V in the culture system
				• Potential model to study host–microbe and host–material interactions
Miller *et al.* 2002 (ref. 94)	NA	Primary gingival fibroblasts in type I collagen matrix	TEM, zymography, western blotting, flow cytometry, live/dead staining, histology	• Matrix remodelling with expression of MMPs and TIMPs
				• Wound healing model
Oberoi *et al.* 2018 (ref. 95)	NA	Primary gingival fibroblasts and periodontal ligament fibroblasts in 3D agarose gels		• Both fibroblasts show ability to form rod shaped microtissues and have contractile behavior
Makkar *et al.* 2022 (ref. 96)	*S. mitis*; *S. oralis*	Primary gingival fibroblasts and periodontal ligament fibroblasts in 3D fibrin-based matrix	Confocal reflectance microscopy, IF, FISH, ELISA	• Denovo deposition of collagen 1 by fibroblasts
	*F. nucleatum* (planktonic and biofilm state)			• Differential immune response by primary and intermediate colonizers to TLR agonists and oral microbes
				• Spatiotemporal colonization of CTEs by primary colonizers.
				• Gingival CTEs secreted higher IL6 and periodontal CTEs secreted higher IL8
Makkar *et al.* 2022 (ref. 22)	*S. mitis*; *S. oralis*; *F. nucleatum*	Primary gingival fibroblasts and microvascular endothelial cells in a 3D fibrin-based matrix (vascularized gingival connective tissue equivalents)	Confocal reflectance microscopy, immunostaining, FISH, ELISA, viability, migration assay	• Gingival CTEs with mature microvasculature
	*A. actinomycetemcomitans* serotype-b,c			• Spatiotemporal colonization of vascularized CTEs by early colonizers and vascular invasion by intermediate and late biofilm colonizers
				• Primary and intermediate colonizers polarize macrophages to anti-inflammatory state and late colonizers polarize macrophages to pro-inflammatory state

### 3.1. Organotypic cultures strategies

3D organotypic cultures range from simple unicellular to multicellular reconstructed gingival/oral mucosal epithelium-only models, connective tissue equivalents (lamina propria equivalents) to more complex full-thickness gingival/oral mucosal tissue equivalents. The complexity and physiological relevance of these models can be further increased by incorporating biological, mechanical, and structural cues to recreate multiple aspects of the native tissue microenvironment and architecture.

**Fig. 3:**
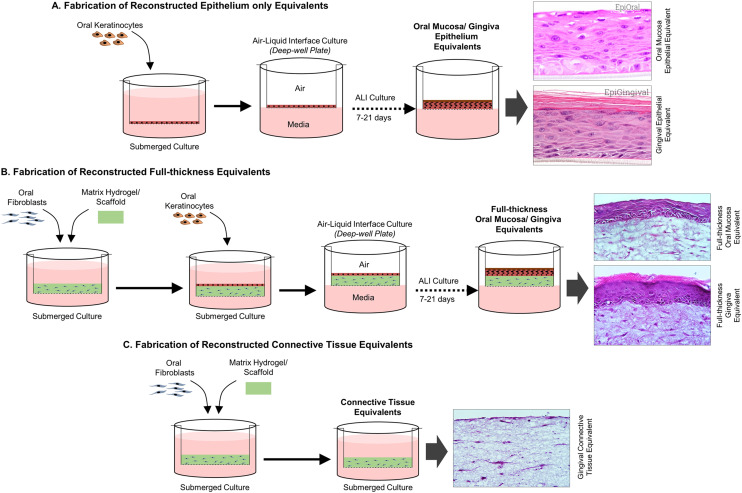
Contribution of organotypic cultures to study periodontal host–microbe interaction. (A) Fabrication of reconstructed epithelial-only equivalents where oral/gingival keratinocytes are seeded on a Transwell as submerged culture and shifted to an air–liquid interface which facilitates epithelial stratification and differentiation. (B) Fabrication of reconstructed full-thickness oral/gingival equivalents which employs casting of a hydrogel enmeshed with stromal cells on a Transwell culture apparatus followed by seeding of keratinocytes as submerged culture and air–liquid interface to generate stratified and fully differentiated tissue equivalents. (C) Fabrication of gingival connective tissue equivalents which involves casting of hydrogel with stromal cells followed by submerged tissue culture.

Reconstructed epithelium-only models are fabricated by seeding keratinocytes directly on a porous membrane or on top of an acellular collagen matrix on a culture insert, followed by culture at air–liquid interface (ALI) to induce stratification and differentiation^20^ (Fig. 3A). This method effectively simulates the stratified squamous epithelium found in oral mucosal and gingival tissues, providing a barrier that is critical for mimicking *in vivo* conditions. The fabrication of organotypic, full-thickness models is similar, but includes the incorporation of fibroblasts within a matrix such as collagen^18,21^ or fibrin^97–99^ to represent the connective tissue (lamina propria) component beneath the epithelium (Fig. 3B). In these models, epithelial cells (*i.e.*, keratinocytes) and connective tissue cells (commonly fibroblasts) interact through epithelial–mesenchymal cross-talk, which regulates epithelial morphogenesis, phenotype, and barrier function.^38,96,100,101^ The terms reconstructed human oral mucosa equivalents (RhOME) and reconstructed human gingiva equivalents (RhGE) are broad terms used to represent both epithelium-only and full-thickness oral mucosal and gingival equivalents respectively. These reconstructed tissue equivalents have been utilized increasingly for basic and translational research including biomaterial compatibility, toxicity, and safety assessments,^97,102–104^ implant-soft tissue interface studies,^102,105,106^ host–microbe interactions,^16,17,20,21,64,65,107,108^ and cancer biology.^109,110^

Additionally, connective tissue-only models (also termed lamina propria equivalents) (Fig. 3C) comprising of its cellular and extracellular components have been utilized to understand the impact of interstitial fluid transport,^24^ fibroblast heterogeneity^96^ and immune cell polarization on innate immune responses against health and disease-associated oral bacteria.^22^ These models help in studying the specific roles of connective tissue components in periodontal health and disease.

### 3.2. Host–microbe co-cultures strategies on 3D organotypic cultures

**Fig. 4:**
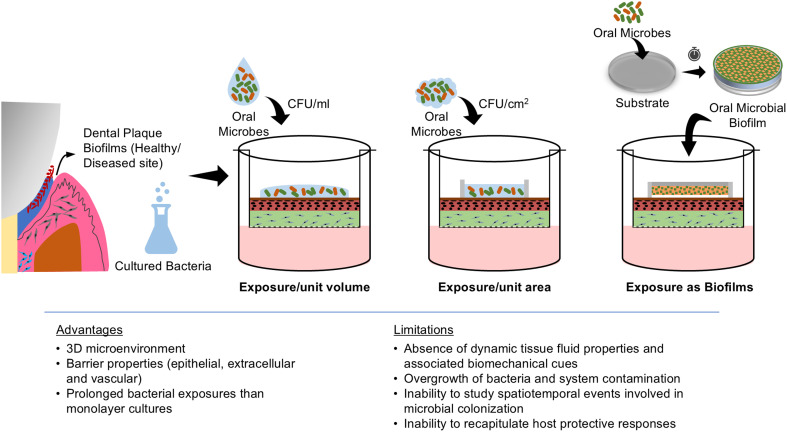
Host–microbe interaction strategies using organotypic cultures. The bacterial biofilm can be exposed in its native state or dissociated state. The dosage of bacterial exposure can be defined by volume (CFU mL^−1^), surface area of the tissue construct (CFU cm^−2^) or exposure of biofilms of known CFUs grown on substrates. The key advantages and limitations of organotypic models for periodontal host–microbe interaction are listed.

Various strategies have been employed to investigate the microbial interactions with gingival/periodontal cells and tissues *in vitro*. In monolayer cultures, host–microbiome studies typically involve exposing host cells to microbes in planktonic states using MOI or CFU ml^−1^.^57,69,111^ In contrast, 3D organotypic tissues exhibit barrier properties offered by both the epithelium and the connective tissue matrix, providing the opportunity for bacterial challenge in both planktonic and biofilm states. Bacterial exposure in these models is typically based on CFU per unit volume of media or CFU per unit surface area of the tissues^16,17,21,22,85,96^ (Fig. 4). Alternatively, biofilms grown on various substrates such as coverslips, hydroxyapatite discs, enamel/tooth slices, and/or implant surfaces are directly placed on the surface of the organotypic cultures (with a spacer) mimicking the natural host–microbial contact and interactions^86^ (Fig. 4).

The ability to challenge the organotypic cultures with both live planktonic and biofilm bacteria allows for flexibility and a physiological recapitulation of the host–microbiome interface of native gingival and periodontal tissues. Mono-species or multi-species commensal and pathogenic biofilms developed from standard bacterial strains or plaque samples derived from human donors can be used over the organotypic cultures.^16,17,21^ This exposure strategy has enabled the study of microbial adhesion, invasion, and biofilm formation under conditions that closely mimic the *in vivo* environment. Through the interaction of biofilms with the epithelial and connective tissue equivalents, studies have demonstrated the impact of barrier integrity, dynamics of immune cell recruitment, cytokine production, and the overall tissue-level inflammatory response to health and disease-associated oral bacteria.^16,17,21,22,85,96^

While studies have demonstrated the application of organotypic cultures to mimic the interactions between symbiotic and dysbiotic biofilms and gingival tissues, other microenvironmental features of the gingival/periodontal-microbe interface need to be recapitulated. From the perspective of microbial microenvironment, most of the Gram-negative bacteria colonizing the periodontal biofilm are strict anaerobes.^7,112^ Culture of gingival and oral organotypic tissue equivalents under anaerobic conditions could hamper cellular health and viability. Although studies have shown that the viability of bacteria is minimally hampered by short-term (up to 4 hours) transitions from anaerobic to aerobic environments,^9,113^ long-term studies require novel strategies to co-culture the host and microbial components under respective gaseous requirements. Custom-designed bioreactors and microphysiological systems that allow recapitulation of aerobic–anaerobic interface could help resolve these challenges. Such systems have been successfully developed and used to simulate the aerobic–anaerobic interface between gut epithelial cells and intestinal microbes, where gut tissues and microbial sustenance have been achieved under hypoxic and anoxic microenvironments.^114,115^

Secondly, bacterial colonization on oral tissues is spatiotemporal in nature, where primary colonizers bind to a receptive surface followed by further colonization of intermediate and late biofilm colonizers.^116^ This sequence of events, transitioning from a symbiotic to a dysbiotic biofilm state, has profound implications in modulating the host's innate immune responses, homeostasis, disease initiation, and progression. Recapitulating the natural progression of biofilm transition events on 3D organotypic culture models would provide a more accurate representation of the host–microbial interface and insights into this interaction.^96^ Developing strategies for long-term co-culture of 3D organotypic models and bacterial biofilms can help in recapitulating the biofilm transitions and studying long-term interactions between host tissues and microbes.

Another important event in disease progression and oral-systemic influences of periodontal disease is the connective tissue invasion by periodontal pathogens and systemic dissemination of bacteria and/or their byproducts *via* vascular invasion.^7,117–119^ Studies using 3D gingival epithelial and vascularized gingival connective tissue equivalents have shown the potential to investigate invasion of biofilm bacteria into the stratified epithelium,^19,80^ connective tissue matrix,^22,96^ and microvasculature.^22^ These advancements could help bridge the gap between *in vitro* studies and the complex *in vivo* environment, offering deeper mechanistic insights into periodontal disease pathogenesis, oral-systemic links, and development of periodontal therapeutic approaches.

### 3.3. Methods to evaluate outcome of periodontal host–microbe interactions in 3D organotypic cultures

Evaluating the outcomes of periodontal host–microbe interactions in 3D organotypic cultures involves various sophisticated techniques to capture the intricate details of these interactions. Given the complex 3D structure and architecture of organotypic models, several methodologies are employed to analyze the extent and nature of tissue responses to microbial exposure.

**Fig. 5:**
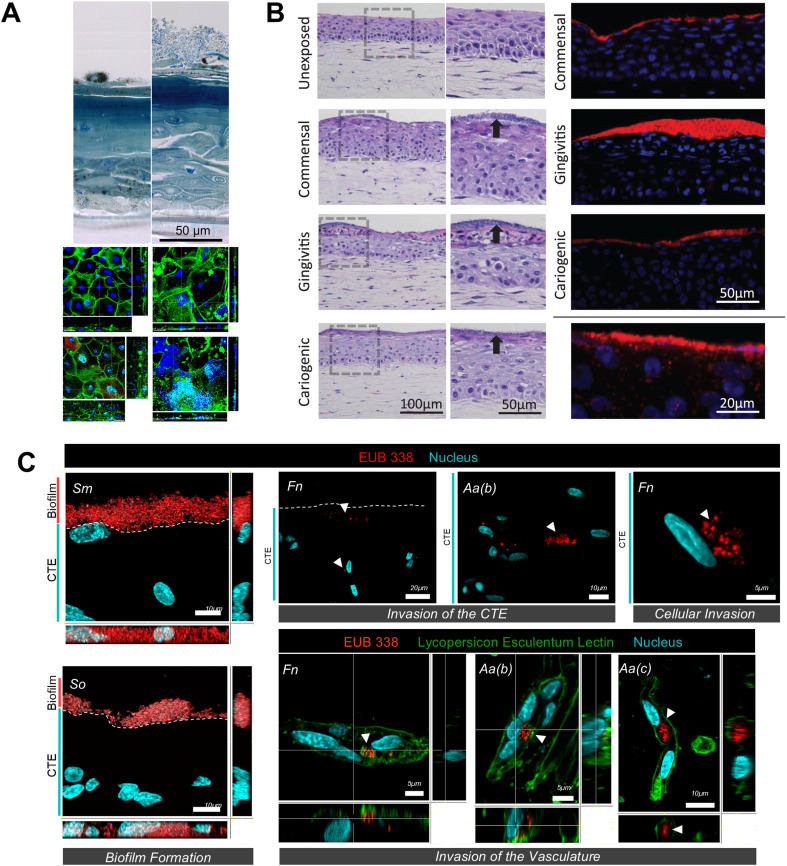
Visualization of host–microbe interaction using organotypic cultures. (A) Histological features of the epithelium only organotypic cell culture model of the oral mucosal showing multilayered stratified epithelium and visualization of oral microbes above it. (B) Histology of the full thickness gingival equivalent showing the presence of epithelium and connective tissue equivalent compartments and visualization of oral biofilms growing on top of the tissue equivalent using HE staining and all bacteria FISH probe EUB338. (C) Images of tissue sections showing well-defined biofilm formation by biofilm colonizers on micro-vascularized connective tissue equivalent as well as their tissue and vascular invasion. Figure panels A, B, and C are adapted from ref. 86 with permission from Elsevier, ©2014, ref. 21 under the terms of the CC-BY license, and ref. 22 with permission from IOP Publishing, ©2023 respectively.

Histological analysis has been a commonly employed method to visualize both the tissue architecture and bacterial biofilm.^16,18,20^ This provides detailed imagery of tissue structure, epithelial morphogenesis, enabling the assessment of tissue invasion and damage following exposure to bacterial biofilms (Fig. 5). Histological staining can be complemented with immunohistochemistry and immunofluorescence techniques to detect the expression of specific proteins, including barrier proteins, cytokeratins, cell proliferation markers, and connective tissue components, before and after microbial exposure.^16,21,82,120^ A study utilizing reconstructed human gingival epithelium demonstrated the symbiotic role of oral commensals on morphogenesis and innate immune response of gingival tissues.^17^ The study observed an increase in gingival epithelial thickness and expression of proliferation markers Ki67 after prolonged exposure to biofilms derived from healthy subjects.

Monitoring the viability of the tissue equivalents post-infection is critical. Time-dependent assessment of lactate dehydrogenase (LDH) released into culture media and MTT assays on fixed tissues are commonly used to evaluate cellular damage and tissue viability after microbial exposure.^83^ Additionally, histological sections of fixed tissues can be analyzed using the TUNEL assay to detect caspase-positive cells, which provides visualization and quantification of epithelial and connective tissue cells undergoing apoptosis triggered by bacterial exposure, its by-products and oral-care formulations.^30,121^

Visualization of microbial adhesion, colonization, invasion, and proliferation is essential to understanding microbial interaction with the host tissues. Fluorescence *in situ* hybridization (FISH) with bacteria-specific probes, combined with confocal microscopy, allows for the detection and visualization of microbial colonization on the surface of the tissue equivalents and invasion into the tissue equivalents (Fig. 5B and C).^17,21,22,96^ Gene and protein expression studies provide assessment and quantification of innate immune response of the organotypic tissue equivalents after bacterial exposures. Previous studies on full-thickness gingival equivalents exposed to microbes have demonstrated the use of gene expression studies to measure the expression of transcripts related to antimicrobial peptides (such as β-defensins), various signalling pathways (such as TLRs, MAPK, NFκB), pro- and anti-inflammatory cytokines and chemokines (such as IL-6, IL-8, IL-10, CCL-2, CCL-20).^16,17,105^ Enzyme-linked immunosorbent assay (ELISA), both singleplex and multiplex, are employed for the absolute quantification of pro- and anti-inflammatory cytokines and chemokines secreted by the tissue equivalents, providing insights into the host's innate immune responses to symbiotic and dysbiotic microbiomes.^16,21,63,64^ Further, high-throughput genomic and proteomic analyses can be employed to identify changes in gene and protein expression profiles in host tissues following microbial exposure. This comprehensive approach can uncover novel biomarkers and pathways involved in periodontal disease pathogenesis. Metabolomic profiling of culture media and tissue samples can help elucidate the metabolic changes associated with host–microbe interactions, thus providing a deeper understanding of the metabolic crosstalk between host cells and microbes.

## 4. Periodontal host–microbe interactions: insights from millifluidic & microfluidic systems

The progression from organotypic cultures to millifluidic and microfluidic OoC systems marks a significant advancement in periodontal disease modeling. While organotypic cultures provide valuable insights into cell–cell and cell–matrix interactions within a static environment, they often lack the ability to replicate the dynamic fluidic conditions, biomechanical cues, and oxygen gradients present *in vivo*. These limitations are critical in periodontal research, where the dynamic flow of fluids such as saliva, gingival crevicular fluid, and interstitial fluid, alongside the mechanical forces from activities like chewing and orthodontic tooth movement, play pivotal roles in nutrient transport, pH regulation, oxygen gradient, biofilm formation, and modulation of host tissue responses.

**Fig. 6:**
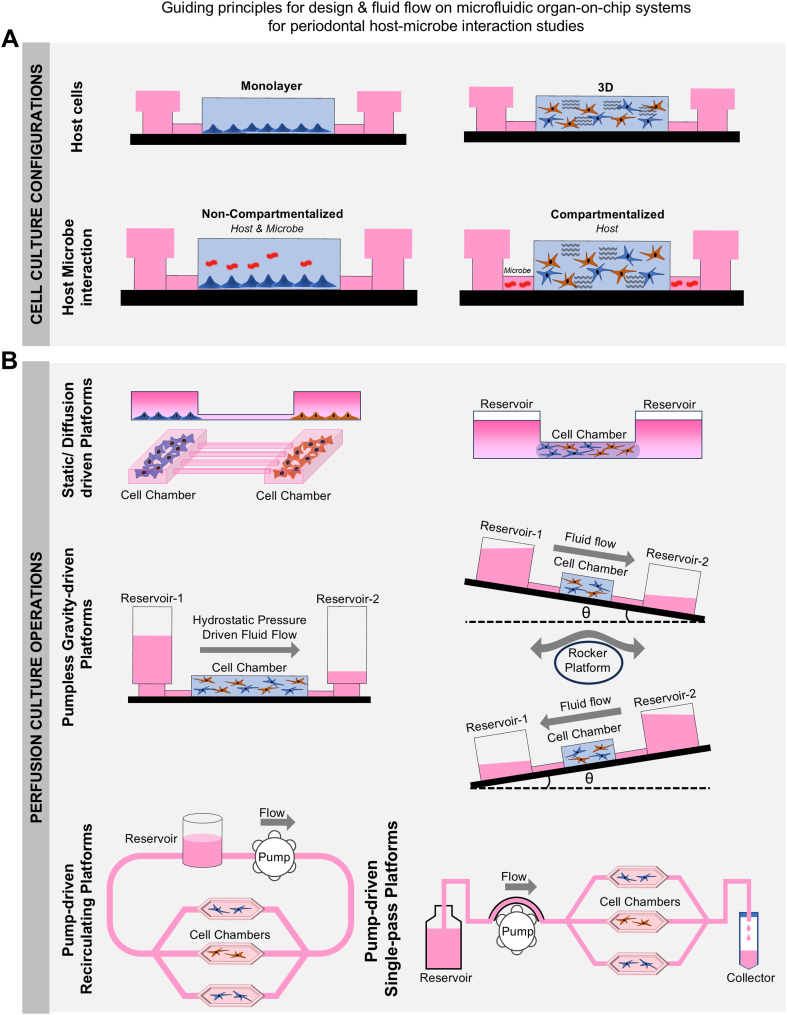
Principles for organ-on-chip design and fluid control for periodontal host–microbe interaction. (A) Host cells can be cultured on fluidic devices as monolayers or in a three-dimensional matrix. The device design dictates the interaction of the microbes with the host cells. The interaction between the host cells and the microbiome can be direct or compartmentalized *via* interconnected sections in the device. This configuration closely recapitulates the periodontal space where the bacterial biofilm and gingival tissue are in close contact with each other, however, have their individual compartments and unique microenvironment essential for their sustenance. (B) Media perfusion is one of the unique capabilities of organ-on-chip devices which facilitates active transport of fresh nutrients for host and microbe compartments as well as continuous removal of metabolic waste. It also plays a crucial role in interstitial flow-induced mechanotransduction and mimicking of fluid flow-induced host protective responses *via* gradient of cytokines and chemokines. The fluid flow on these devices can be regulated *via* hydrostatic pressure-based pump-free systems which can work either by altering media column height in the fluid reservoir or placing the devices on a rocker platform which aids in developing the gradient. Active pumping using an external pump is another method to drive fluid flow where a peristaltic pump or syringe pump can be connected to the device and recirculatory or single-pass fluid operations can be initiated and controlled.

OoC systems represent a sophisticated amalgamation of advanced cell culture, tissue engineering, and bioengineering techniques. These systems incorporate intricate design features enabling geometrical confinement, cell patterning, controlled fluid flow, microenvironmental regulation, sensor integration, and downstream on-chip and off-chip readout capabilities.^122–125^ Microchannels and microchambers within these devices allow manipulation of fluid flow, nutrient delivery, elimination of metabolic waste, and the collection of cell secretome, mimicking the role of vascular and interstitial tissue fluid flow in native tissues (Fig. 6). These capabilities enable the emulation of key functional units of human tissues and organs, crucial for studying tissue–tissue, tissue–biomaterial, and tissue–microbe interfaces under conditions that closely resemble *in vivo* microenvironments.^122,126–128^

These features of the OoC systems provides the opportunity to build physiologically-relevant platforms, facilitating a deeper understanding of host–microbe interactions, biofilm development, and the overall pathogenesis of periodontal disease. In the recent years, this has to an increasing adoption of microfluidic OoC and microphysiological systems to replicate oral and dental barrier tissues.^13,14,129,130^ Fluidic systems is gaining attention in periodontal research allowing the simulation of complex microbial communities and dynamic microenvironments typical of oral biofilms.^131–134^ These systems facilitate the study of biofilm formation and maturation under controlled conditions, providing insights into microbial interactions, spatial organization, and responses to therapeutic interventions. Further, by incorporating keratinocytes, fibroblasts, and immune cells, with microbial pathogens and oral-care formulations, OoC systems have provided new insights into inflammatory responses and tissue barrier function.^24,27–31,135^ Moreover, the ability to regulate fluid flow (such as saliva, interstitial fluid, and oral-care products) and mechanical forces has enhanced the physiological relevance and insights on tissue behavior and microbial interactions.^24,27,29,30,136^ Furthermore, these technologies have facilitated the evaluation of dental materials and implants within realistic oral tissue microenvironments, assessing biocompatibility, cellular viability, and tissue-level responses under mechanical and dynamic fluid flow conditions.^25,31,97,135,137^ These systems also have offered insights into the progression of oral cancer, including tumor growth dynamics, invasion into surrounding tissues, and responses to radiotherapy, chemotherapy, and oral mucositis.^138–140^

OoC platforms for dental and craniofacial applications developed over the past decade encompass a diverse array of models, including tooth-on-chip,^25,26,97^ gingiva-on-chip,^28–30,141^ gingival crevice-on-chip,^24^ periodontal ligament-on-chip,^136,142^ oral mucosa-on-chip,^31,135^ oral mucositis-on-chip,^139,140^ oral cancer-on-chip,^143^ pulp-like tissues on-chip,^144^ to salivary gland-on-chip.^145^ Each of these platforms tailored to simulate specific aspects of dental and oral physiology. By providing a comprehensive platform for studying microbial, material, and host dynamics, these technologies are revolutionizing our understanding of dental and oral tissues across health and disease states. Specific applications of these fluidic platforms to study oral biofilms and host–microbe interactions are summarized in Table 4.

**Table 4:** Summary of studies on the application of microfluidic and millifluidic based platforms to study oral biofilms and host–microbe interactions

Authors	Aim	Chip design, materials & flow type	Cell/bacterial type	Culture parameters	Assays
Foster and Kolenbrander 2004 (ref. 146)	Examine the development of multispecies oral biofilm in a saliva-conditioned flow cell	Single-channel flow cell, high-density polyethylene block	Bacteria: *S. gordonii*, *A. naeslundii*, *V. atypica*, *F. nucleatum*	25% sterile human saliva	FISH, confocal microscopy, Syto 59 nucleic acid staining, live/dead viability assay
		Flow: peristaltic pump		Flow rate: 200 μL min^−1^	
Eun & Weibel 2009 (ref. 147)	To investigate adhesion and formation of geometric controlled microbial biofilm arrays on different substrate	Multichannel with PDMS stencil array for patterning	Microbes: *P. aeruginosa*, *B. subtilis*, *S. epidermidis*, *C. albicans*, *E. coli*, *V. fischeri*	Different media depending on the microbe	Biofilm growth, live/dead staining, confocal microscopy, fluorescence microscopy, SEM, interferometry
		Flow: syringe pump			
Janakiraman *et al.*, 2009 (ref. 148)	Develop a mathematical model of quorum sensing and investigate biofilm growth in microfluidic chambers	Single-channel microfluidic chamber, PDMS	Bacteria: *P. aeruginosa*	LB medium, oxygen levels controlled, acyl-HSL as quorum sensing molecule	Quorum sensing (QS) model, biofilm thickness measurement, computational fluid dynamics
		Flow: syringe pump			
Goeres *et al.*, 2009 (ref. 149)	Protocol to grown biofilms under low shear at the air–liquid interface	4-Channel drip flow biofilm reactor, glass coupons	Bacteria: *P. aeruginosa*	Bacteria culture media	Viable plate counts
		Flow: peristaltic pump		Flow rate: 0.8 ml min^−1^	
Zainal-Abidin *et al.*, 2012 (ref. 150)	Investigate protein essential for bacterial interactions in a polymicrobial biofilm	Single-channel flow cell, glass coverslip	Microbes: *P. gingivalis*, *T. denticola*, *T. forsythia*	Oral bacteria growth medium	FISH, confocal microscopy, real-time PCR, LC-MS/MS, SEM
		Flow: peristaltic pump		Flow rate: 3 mL h^−1^	
Ali Mohammed *et al.*, 2013 (ref. 151)	To characterize extracellular polymeric matrix components and test DNase I and proteinase K effects on biofilms	Three-channel flow cell, glass cover slip	Bacteria: *F. nucleatum*, *P. gingivalis*	Bacterial growth medium	Biomass thickness, confocal microscopy, carbohydrate and eDNA yield
		Flow: peristaltic pump		Flow rate: 3.3 ml h^−1^	
Nance *et al.*, 2013 (ref. 152)	Develop a high-throughput microfluidic system to evaluate the effectiveness of antimicrobials against multi-species oral biofilms grown in human saliva	BioFlux microfluidic plates, with 48 wells and 24 channels	Dental plaque biofilm	Cell-free saliva	Biofilm viability (live/dead), confocal microscopy, bacterial tag-encoded FLX amplicon pyrosequencing, Comstat analysis
		Flow: pneumatic-driven		Flow rate: 19 ml h^−1^	
Bao *et al.* 2015 (ref. 23)	To develop a model of a periodontal pocket using a perfusion bioreactor system	Cellec Biotek AG perfusion bioreactor	Host cells: immortalized epithelial cells (HGEK-16), fibroblasts (FB-16), and a monocytic cell line perfused through 3D collagen scaffold into the bioreactor	Defined keratinocyte SFM	RT-PCR, quantification of cytokine secretion, Masson's trichrome staining, SEM
			Microbes: *P. gingivalis*, *P. intermedia*, *A. actinomycetemcomitans* JP2, *C. rectus*, *V. dispar*, *F. nucleatum*, *S. oralis*, *T. denticola*, *A. oris*, *S. anginosus*, *T. forsythia*	Bacterial specific media	
Lam *et al.* 2016 (ref. 153)	To investigate the growth of streptococci and *Fusobacterium nucleatum* in biofilm state under variable dissolved gases and sucrose concentration	Multiarray, PDMS	Biofilm from healthy subjects	Artificial saliva	FISH, biomass quantification, live/dead assay
Gashti *et al.* 2016 (ref. 134)	To understand the chemical and hydrodynamic effects on pH changes in oral biofilm	Single chamber, PDMS	*S. salivarius*	Media: unbuffered Luria Bertini broth	Confocal microscopy, pH measurement
				Flow rate: variable	
Rath *et al.* 2017 (ref. 131)	To investigate accumulation of biofilms on titanium implant surface	Single chamber, polyaryletherketone	*S. gordonii*, *S. oralis*, *S. salivarius*, *P. gingivalis*, *A. actinomycetemcomitans*	Bacterial specific media	Confocal microscopy with dead live assay, mean biofilm thickness
				Flow rate: 100 μL min^−1^	
Rahimi *et al.* 2018 (ref. 31)	Microfluidic oral mucosa model-on-a-chip with proof of concept host–microbe interaction studies	3 parallel channels communicating *via* micropillars, PDMS	Host cells: fibroblast cell line-laden collagen matrix, followed by a layer of keratinocyte cell line (Gie-No3B11)	Bacterial specific media	Confocal microscopy, TEER, live/dead assay
		Flow: tension-driven	Microbes: *Streptococcus mutans*	Prigrow III and IV media for culturing keratinocytes and fibroblasts respectively	
Luo *et al.* 2019 (ref. 132)	To quantify the architecture of oral biofilms in antibiofilm interventions	Single chamber, PDMS	Bacteria from healthy subjects	Saliva from healthy subjects	Confocal microscopy with dead live assay, viability
		Flow: syringe pump			
Kristensen *et al.* 2020 (ref. 133)	To understand the impact of stimulated saliva flow on pH changes in dental biofilms	Single chamber, 3D printed resin	Biofilms from healthy subjects	Stimulated saliva from healthy subjects	Confocal microscopy, pH measurement
		Flow: syringe pump *via* tubing			
Rodrigues *et al.* 2021 (ref. 26)	Recapitulation of biomaterial–biofilm–dentin interface on a microfluidic device	Double-chambered and channeled PDMS chip with a groove to fit dentin slices	*S. mutans* monospecies biofilm	Odontogenic media for host cells and buffered tryptone yeast extract broth for bacteria	Viability, immunostaining, ph measurement, ELISA
Jin *et al.* 2022 (ref. 28)	Model recapitulating periodontal soft tissue (epithelial–endothelial interface)	Parallel channel overlying each other and separated by porous membrane, PDMS	Host cells: human gingival epithelial and umbilical vein endothelial cells	Keratinocyte growth media (epithelial cells)	Confocal microscopy, immunostaining, ELISA
		Static conditions	Microbes: LPS and TNF-α treatment	EBM-2 media (endothelial cells)	
				Induction of inflammation with LPS and TNF-α treatment	
Ghesquière *et al.*, 2023 (ref. 154)	To develop and characterize a five-species periodontal biofilm model cultured under dynamic conditions	Drip flow biofilm reactor, glass slide	Bacteria: *S. gordonii*, *S. oralis*, *S. sanguinis*, *F. nucleatum*, *P. gingivalis*	Supplemented, modified BHI media under anaerobic and aerobic conditions	Biomass thickness, confocal microscopy, metabolite profiling, PCR
		Flow: peristaltic pump		Flow rate: 0.11 ml min^−1^	
Makkar *et al.* 2023 (ref. 24)	To recapitulate the cellular, structural and fluid flow properties of the gingival connective tissue including the flow of gingival crevicular fluid (GCF)	Parallel chambers communicating with channels *via* micropillars, PDMS	Host cells: primary gingival fibroblasts in 3D fibrin-based matrix	Chemically defined culture media, human saliva for adhesion of microbes seeded in crevicular channel	Whole-mount immunostaining, confocal microscopy, fluorescence recovery after photobleaching, ELISA, LDH cytotoxicity assay
		Flow: hydrostatic pressure-driven (pumpless)	Microbes: *S. oralis* (ATCC 35037), *F. nucleatum* (ATCC 25586) and TLR2 agonist	Impact of simulated GCF on modulation of host innate immune response	
Adelfio *et al.* 2023 (ref. 27)	To develop a gingival bioreactor which supports long term culture of gingival equivalents *in vitro* and host microbe interaction studies	PDMS replica molding of a 3D printed model of gum-tooth unit. Silk solution used to generate porous extracellular matrix	Host cells: primary oral keratinocytes and fibroblasts	Serum free defined media condition, artificial saliva	Viability, gene expression, immunostaining, SEM, ELISA
		Flow: peristaltic pump	Microbes: LPS (*P. gingivalis*)		
Gard A. L. *et al.* (2023) (ref. 141)	High throughput microfluidic model of human gingiva for host microbe interaction studies and biomaterial testing	PREDICT96 prefabricated system	Host cell–donor matched oral keratinocytes, oral fibroblasts, and commercially sourced dermal microvascular endothelial cells	Proprietary low calcium defined media	TEER, ELISA, immunostaining
		Flow: on-chip micropumps (recirculation)			
Ramachandra S. S. *et al.* (2024) (ref. 155)	Bioreactor system to assess antimicrobial efficacy on polymicrobial periodontal biofilms	Commercially available CDC bioreactor system	Subgingival dental plaque from patients with stage III periodontitis	Nutrient broth with and without antibiotics	Viability, SEM, RT-PCR
		Flow: peristaltic pump			
Svanberg *et al.*, 2024 (ref. 142)	Develop vascularized periodontal ligament model to emulate the physiopathology of human periodontal ligament and study early stages of inflammatory diseases	1–3 parallel chambers spanned by channels. PDMS	Host cells: human umbilical vein endothelial cells and patient-derived periodontal ligament cells	Endothelial growth medium with and without VEGF supplements	Perfusability, permeability assays, immunofluorescence staining, confocal imaging, second harmoinic imaging, cytokine detection, ELISA
		Flow: peristaltic pump	Bacteria: LPS	Flow rate: 60 μl h^−1^	

### 4.1. Fluidic design, material selection and fabrication strategies

The design and fabrication of dental and periodontal OoC systems require careful consideration of materials and methodologies to replicate the complex microphysiological environment of oral tissues. The design characteristics of an OoC system significantly impact its functionalities, potential applications, and inherent limitations.^156^ The ability to tailor the design features of the OoC systems enable the replication of the microphysiological conditions of tissues and their interactions with both internal and external environments. Fig. 6 provides an overview of the fluidic design and control. For a comprehensive overview of various design features, materials, and strategies for the fabrication of OoC systems, readers are directed to detailed reviews in the literature.^13,15,156–158^

In the context of oral biofilms and periodontal health and disease-related applications, geometrical compartmentalization, cell patterning, and controlled fluid flow and oxygen gradients are key features that enable overcoming the limitations of organotypic cultures. Materials used in the construction of OoC devices for periodontal applications must offer cellular and microbial compatibility, optical transparency, and appropriate gas permeability to support both aerobic and anaerobic cultures. Poly(dimethylsiloxane) (PDMS) is frequently used due to its optical clarity, elasticity, and gas permeability, making it ideal for imaging, mechanical stimulation,^29^ and the cultivation of aerobic bacteria and host (gingival/periodontal) cells.^24–26,31,139,142^ Thermoplastic materials like poly(methyl methacrylate) (PMMA), polycarbonate (PC) are other popular materials commonly used in OoC devices for dental applications due to their transparency and gas-impermeable properties.^30,97,136,144^ These materials can be 3D printed,^133^ assembled from thermal bonding of pre-polymerized sheets,^30,97^ or mass-produced through injection molding.^144^ The gas-impermeable nature of thermoplastics is advantageous for culturing anaerobic periodontal bacteria, while the gas-permeable feature of PDMS is beneficial for the growing host cells and aerobic bacteria.

Managing fluid flow within these devices is critical for simulating the oral environment. Various fluid control methods are employed to perfuse media through the OoC device and to emulate the fluid flow and microenvironmental features of the oral, gingival, dental, and periodontal tissues. Each fluid control method has its unique set of advantages and limitations, and the choice greatly depends on whether a one-pass or a recirculatory flow is intended (Fig. 6). Passive methods, such as hydrostatic pressure and gravity-driven systems, offer simplicity and ease of use, though they may lack precise control over flow rates (references). External peristaltic, syringe, or pneumatic pumps are frequently used in dental and periodontal OoC systems to manage fluid flow.^27,30,31,136,137^ These active systems provide precise control, facilitating the replication of physiological flow conditions and shear stress encountered in the oral cavity. However, connecting these external systems can be intricate and challenging for non-expert users, potentially increasing the chance of contamination and bubble formation, thereby limiting their wider use. Alternatively, fluid flow in dental and periodontal OoCs have been managed using simpler passive levelling-based methods such as rocker platforms, hydrostatic pressure, or tension-driven pumpless flow designs.^24–26,31^ While relatively straightforward, drawbacks include bidirectionality of flow when using rocker platforms and variable or continuously declining flow rates in hydrostatic pressure or tension-driven pumpless systems. Alternatively, high-throughput OoC systems with integrated pumps and biosensing capabilities like PREDICT96 have been employed to culture vascularized gingival tissues under healthy and inflamed states for the development of novel periodontal therapeutics.^141^

### 4.2. Application of fluidic devices for oral biofilm development

**Fig. 7:**
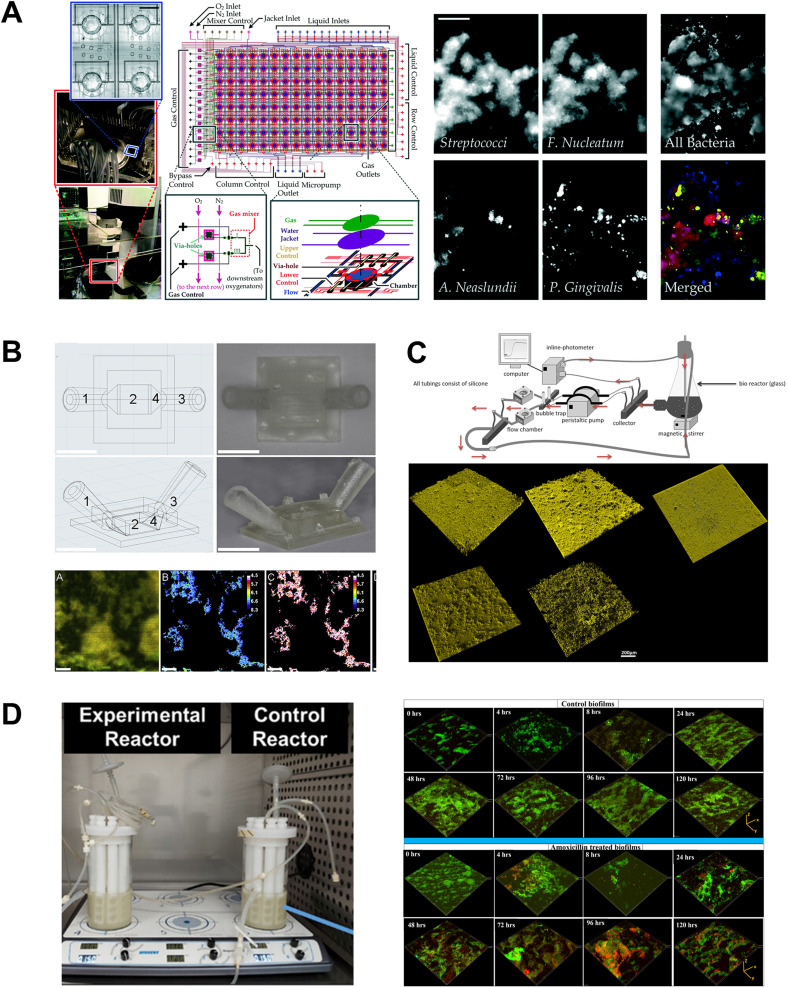
Fluidic devices for the culture of periodontal bacterial biofilms. Oral biofilm formation is a precursor to dental and periodontal microbial diseases and fluidic systems have been developed to understand this in isolation. (A) A high-throughput microfluidic device designed to regulate various microbial microenvironmental factors, enabling the study of spatial distribution, thickness, and viability of oral biofilm colonizers. Similarly, the flow cells in (B and C) were developed to cultivate biofilms under shear-controlled conditions, allowing for real-time monitoring of pH, biofilm growth dynamics, and viability. (D) Commercially available bioreactor system employed to grow periodontal polymicrobial biofilms to understand the antimicrobial efficiency of clinically relevant dosage of antibiotic *via* viability imaging. Figure panels A–D are adapted from ref. 153 with permission from The Royal Society of Chemistry, ©2016, ref. 133 with permission from Elsevier, ©2020, ref. 131 under the terms of the CC-BY license, and ref. 155 under the terms of the CC-BY license, respectively.

Engineered flow devices integrated with fluid delivery systems have been utilized to investigate biofilm adhesion, formation, maturation, and interspecies interactions under dynamic flow conditions (Fig. 7A–D). Shear forces generated by the movement of saliva within the oral cavity play a crucial role in biofilm development, which influences spatial organization, nutrient uptake, and surface area of bacterial growth. Flow cell bioreactors and microfluidic platforms have been employed to study the effects of shear forces on biofilm behavior.^150,151^ Drip-flow reactors with inclined channels and gentle continuous flow enable the formation of biofilms along the direction of liquid flow and at air–liquid interface, closely simulating the conditions within the oral cavity.^149,154^ The dripping mechanism offers fluid flow with low shear, which can be controlled by adjusting the inclination of the biofilm surface. Ghesquière *et al.*^154^ developed a multispecies periodontal biofilm model using a drip flow reactor, which allowed for the real-time profiling of biofilm structure, metabolic activity, and insights into how shear forces and fluid dynamics influence biofilm structure and function. Further, they demonstrated its application to investigate the effects of prebiotic treatments like l-arginine on biofilm development under physiologically relevant flow conditions. Flow cell systems have also been used to investigate the inter-species interactions and dynamics within subgingival plaque by allowing continuous cultivation and observation of biofilm dynamics over extended periods. Zainal-Abidin *et al.*^150^ utilized a single-channel flow cell system to study red-complex bacteria, demonstrating that the upregulation of glycine catabolism in *Porphyromonas gingivalis* induced structural changes in the flagella of *Treponema denticola*.

Surface adhesion is another critical aspect of biofilm development on tooth and implant surfaces. Rath and colleagues^131^ utilized a customized flow cell to study microbial colonization on implant surfaces (Fig. 7C). They recirculated a suspension of *Streptococcus gordonii*, *Streptococcus oralis*, and *Porphyromonas gingivalis* at flow speeds mimicking salivary flow rates, allowing them to investigate the dynamics and characteristics of biofilm formation on the implant surface. Eun and Weibel^147^ demonstrated the use of micropatterned PDMS stencils to pattern biofilms on geometrically controlled substrates, enabling precise control over where bacteria could adhere and form biofilms. This method effectively recreated spatially organized biofilm structures. Further, the integration of these micropatterned substrates within microfluidic channels allowed for fine-tuning of fluid dynamics and surface interactions, which are critical in the early stages of biofilm development.

Fluid flow such as the flow of saliva and GCF influences the microenvironmental conditions such as nutrient delivery, dissolved gases, and action of host protective factors which influence biofilm physiology. Kristensen *et al.*^133^ explored the impact of flow conditions on the pH of dental biofilms, wherein donor-derived plaque biofilms were cultured in the presence of simulated saliva in 3D printed flow cells (Fig. 7B). The study showed that biofilms grown in static conditions exhibited a significant decrease in pH levels. In contrast, those maintained under flow conditions exhibited a variable pH gradient throughout the biofilm, similar to natural oral biofilms. Using microfabrication and microfluidic technology, Lam *et al.*,^153^ developed a high-throughput system that enabled long-term bacterial growth and biofilm development (Fig. 7A). This device allowed precise control of various microphysiological parameters of biofilm culture, including nutrient delivery, dissolved gases, and microbial seeding density. Further, Nance *et al.*^152^ used the high-throughput BioFlux microfluidic system to evaluate the effectiveness of antimicrobials against multi-species oral biofilms grown in human saliva. The microfluidic design enabled miniaturization, minimized the amount of saliva needed, and allowed for precise manipulation of fluid flow. Further, the multi-well/multi-channel (24 channels and 48 wells) facilitated high-throughput screening of antimicrobial effect of a wide range of cetylpyridinium chloride on the biofilms.

In summary, the application of fluidic devices in studying oral biofilm dynamics offers significant advantages over traditional static models. These systems provide precise control over fluid dynamics, shear forces, nutrient delivery, and gas concentrations, enabling detailed investigations into the complex behaviors of oral biofilms, and developing effective strategies to manage periodontal health and disease.

### 4.3. Application of microfluidic OoC devices for host–microbe and host–material studies in dental and periodontal applications

**Fig. 8:**
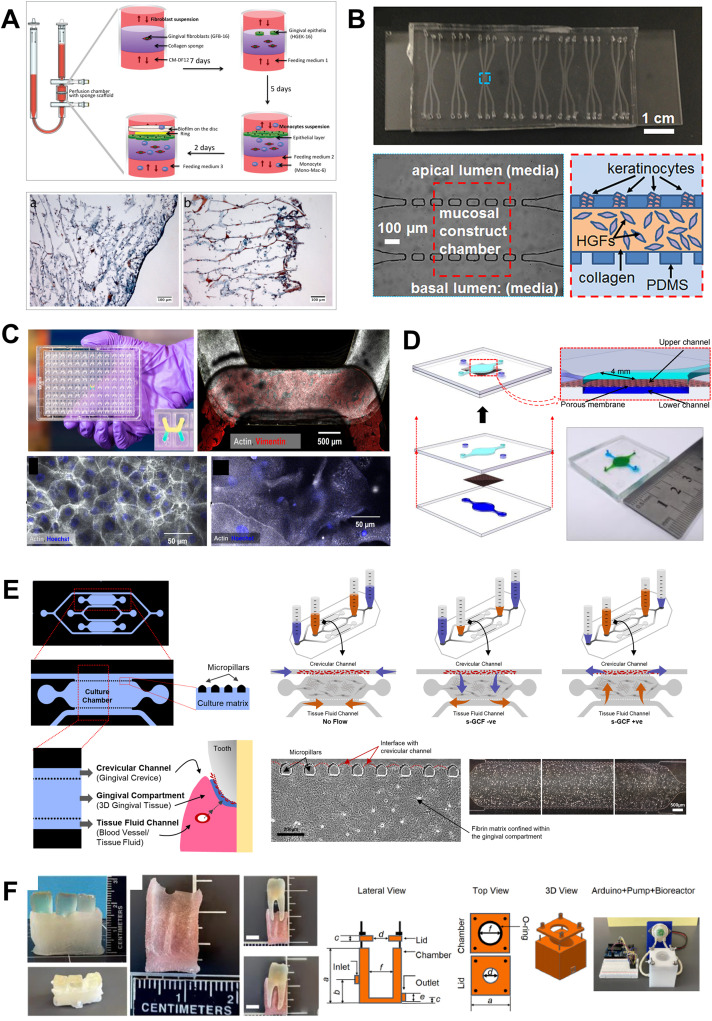
Organ-on-chip systems design configurations. The design characteristics of the OoC devices is guided by the intended application, cell culture configuration, and readouts. This can range from (A) perfusion bioreactor-based millifluidic devices, (B–D) multi-compartmental microfabricated devices and (E) 3D printed molds to generate organ-chip device. (A and E) The bioreactor-based configuration allows the use of a collagen or silk scaffold inside the perfusion chamber where cells are seeded and host–microbe interaction studies are performed. Microfabricated devices used for periodontal host–microbe interaction have been designed using lateral and apical chamber/channel configuration. The lateral chamber channel configuration (B and E) utilizes a post (rectangular or pentagonal) separating the culture chamber from the adjoining channel. The inter-post distance and the chamber height allow loading and containment of hydrogels enmeshed with cells in the chamber. The lateral channels can be used to seed another cell type, media perfusion, or generation of an air–liquid interface. The apical configuration devices (C and D) have a porous membrane dividing the culture chambers which are used for bi-cellular culture, the active flow of media in one channel, and generation of air liquid interface. Figure panels A–F are adapted from ref. 23 under the terms of the CC-BY license, ref. 31 with permission from AIP Publishing, ©2018, ref. 141 under the terms of the CC-BY license, ref. 28 under the terms of the CC-BY license, ref. 24 with permission from Wiley-VCH GmbH, ©2022, and ref. 27 with permission from Acta Materialia Inc, ©2023, respectively.

**Fig. 9:**
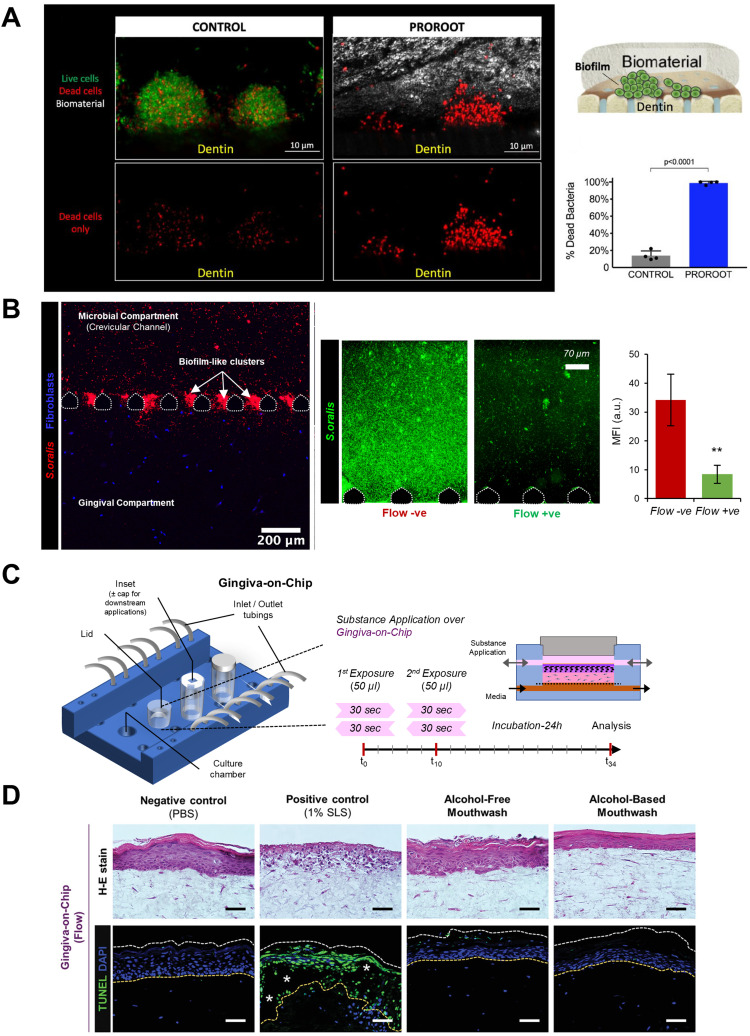
Evaluating host–microbe and host–material interactions on organ-on-chip devices. OoC devices allow spatiotemporal visualization of oral host–microbe–material interactions and understand the impact of antibacterial properties of dental biomaterials and biocompatibility of oral-care products under microphysiological conditions. Can be evaluated on tooth-on-chip platform and gingiva-on-chip respectively. (A) The tooth-on-chip platform allows recapitulation of dentin-pulp complex where human tooth sections are embedded on a PDMS-based device dividing the host cells and bacterial biofilms compartments. Dental bioceramics having antibacterial properties are introduced in the device, contacting the bacterial biofilm and its time-dependent antibacterial effect is evaluated. (B) Long-term host–microbe co-existence *via* compartmentalization, visualization of biofilm formation, and the protective effect of GCF flow towards washing of bacterial biofilms has been studied using the gingival crevice-on-chip. (C and D) gingiva-on-chip allows a perfusion-based culture of full-thickness gingival equivalents where the chip design allows apical access to the epithelial surface allowing the recapitulation of mechanical action of mouth rinse. The tissue equivalents exposed to oral-care formulations can be harvested and histological evaluation performed. Figure panels A–C are adapted from ref. 26 with permission from Sage Publications, ©2021, ref. 24 with permission from Wiley-VCH GmbH, ©2022, and ref. 30 under the terms of the CC-BY license, respectively.

Microfluidic OoC systems have been employed to simulate host–microbe interface, tissue fluid flow, and shear stress, providing valuable insights into host–microbe and host–material interactions in dental and periodontal contexts (Fig. 8 and 9). In some of the earliest studies, Bostanci and colleagues,^23,63^ demonstrated the application of a commercially available perfusion bioreactor system to develop a proof-of-concept model of a periodontal pocket (Fig. 8A). Using a perfused liquid–liquid interface culture of monocytes through a scaffold seeded with fibroblasts, keratinocytes, and multispecies biofilm growing on hydroxyapatite discs, they demonstrated the potential to study the interactions between gingival tissues and complex multi-species microbial biofilms within the periodontal pocket. While the system is bulky, it provided the pioneering impetus for the application of fluidic systems for recapitulating the complex host–microbe interactions *in vitro*.

Raub and colleagues^31^ fabricated a three-channel microfluidic oral mucosa-on-a-chip device seeded with keratinocytes and gingival fibroblasts enmeshed in a collagen matrix (Fig. 8B). In a series of studies, the oral mucosa-on-a-chip platform was utilized to investigate responses to dental materials and microbes.^31,135^ Further, the platform was used to model oral mucositis-on-a-chip following radiation and chemotherapy^139^ and investigate the possible cellular and molecular mechanisms of oral mucositis and potential therapeutic targets for recovery of oral mucositis.^140^

The interstitial fluid flow through the gingival connective tissue contributes to the formation of GCF, a physiological fluid that exhibits characteristics of both transudate and inflammatory exudate depending on whether the crevicular wall is in a healthy or inflamed state.^35^ The GCF helps in host protection by washing away the bacteria, its by-products, and toxins from the gingival crevice. Makkar *et al.*,^24^ developed a microfluidic gingival crevice-on-chip featuring an elongated hexagonal microchamber flanked by microchannels to emulate the interstitial and GCF flow through the connective tissue wall of the gingival crevice, and investigated the influence of GCF flow on modulating innate immune responses to oral bacteria (Fig. 8E). Computational fluid dynamics simulations and validation with fluorescence recovery after photobleaching (FRAP) assays, provided insights on the diffusion kinetics of fluorescein-conjugated dextran macromolecules of 10 kDa and 70 kDa sizes. Similarly, using an epithelium-capillary interface-on-a-chip, Jin *et al.*,^28^ demonstrated the endothelial barrier function and selective permeability of the endothelial barrier to 40 kDa and 70 kDa dextran macromolecules (Fig. 8D). These studies provided insights into the macromolecular perfusion characteristics, representing the fluid dynamics of transudate in healthy states and exudate in inflamed or diseased states.

OoC devices have also been instrumental in overcoming the challenges of static culture systems, particularly in achieving long-term host–microbe co-culture of gingival tissues and oral bacteria.^24,27,28,31^ By incorporating compartmentalized sections for host cells and microbes, studies using gingival crevice-on-chip and epithelium-capillary interface-on-a-chip have demonstrated long-term host–microbe co-culture^24^ and simulation of the protective effects of GCF flow such as microbial clearance and modulation of innate immune response against periodontal pathogens^24,28^ (Fig. 9B). Furthermore, using NFκB inhibitor (pyrrolidinedithiocarbamic acid) as a proof-of-concept drug, Jin *et al.*,^28^ demonstrated the potential to recover the decreased barrier integrity following exposure of the gingival tissues to TNF-alpha and LPS on an epithelium-capillary interface-on-a-chip. This highlights the potential application of OoC-based gingival tissue–microbe interface to simulate fluid flow characteristics, enable long-term host–microbe interactions, study gingival inflammation and to screen potential periodontal therapeutics under microphysiological conditions close to the native gingival and periodontal tissues in healthy and diseased states.

As a barrier tissue, the gingiva experiences mechanical forces and shear stress from salivary flow and various activities such as chewing food, tooth brushing, and rinsing mouthwash. Lee *et al.*,^29^ developed a 3D oral epi-mucosa platform, to investigate the role of mechanical stress and matrix stiffness on the epithelial barrier. Their findings indicated that collagen matrices with intermediate stiffness (30 Pa) preserved barrier integrity, while softer (10 Pa) and stiffer (120 Pa) matrices compromised it. Muniraj *et al.*^30^ developed a microfluidic gingiva-on-chip platform to biofabricate full-thickness gingival equivalents and ulcer-on-chip equivalents. The gingiva-on-chip platform demonstrated enhanced epithelial development and barrier functionality when subjected to dynamic flow conditions. Additionally, the gingiva-on-chip and ulcer-on-chip equivalents were employed to investigate the mechanical impact of mouth rinses, demonstrating that oral-care formulations caused greater tissue disruption and cytotoxicity under dynamic conditions compared to static exposure (Fig. 9C and D). Similarly, Adelfio *et al.*^27^ developed a dynamic gingival tissue model replicating the cytoarchitecture and oxygen concentrations of human gingiva (Fig. 8F). Using a custom bioreactor, this model facilitated the replication of physiological shear stress of salivary flow, improved epithelial barrier, and long-term stability of the gingival tissues when challenged with *P. gingivalis* lipopolysaccharide, highlighting its potential for long-term studies on host–pathogen interactions. Gard *et al.*^141^ developed the MOUTH (microfluidic model of oral physiology for understanding tissue health) model which enabled the culture of a multi-layered gingival tissue on a multi-arrayed microfluidic platform, and simulation of healthy and diseased states of gum tissue *in vitro* (Fig. 8C). This model, which co-cultured human primary gingival cells with human microvascular endothelial cells for up to 4 weeks, enabled the investigation of long-term mucosal barrier function and cytokine secretion. Key features of this study included stable TEER over multiple weeks, indicating mature barrier function, and the ability to induce and recover from an inflammatory state. Similarly, the periodontal ligament-on-chip model developed by Svanberg *et al.*^142^ incorporated perfusable vasculature using periodontal ligament cells and endothelial cells within a fibrin-based hydrogel and demonstrated its potential to study changes in extracellular matrix and cytokine production in response to LPS stimulation, and its application to understand early inflammatory events in periodontitis.

From the perspective of the application of OoC systems for host–material and host–microbe interactions on dental tissues and dental caries, Franca *et al.*^25^ pioneered the development of a tooth-on-a-chip model. This horizontally-stacked device enabled real-time tracking of dental pulp cell responses to various dental materials, highlighting their cytotoxic effects and gelatinolytic activity in a model hybrid layer. In a follow-up study by Rodrigues *et al.*,^26^ the tooth-on-chip platform was utilized to evaluate the effects of calcium silicate cements on dental pulp stem cells, showing ProRoot's significant impact on TGF-β release from dentin slices, cell proliferation, and its antimicrobial properties against *Streptococcus mutans* biofilm (Fig. 9A). Similarly, Hu *et al.*,^97^ designed another tooth-on-chip platform (vertically-stacked device) to demonstrate the cytotoxicity of silver diamine fluoride (SDF) on dental pulp cells and the impact of dentin barrier in modulating the permeation of SDF and the subsequent cytotoxic response. A dentin slice, representing the tooth in these OoC systems, serves as a semi-permeable barrier that mimics the dentin-pulp complex. These tooth-on-chip models have enabled the direct visualization of interactions between dental materials and the dentin-pulp complex, as well as the real-time assessment of antibiofilm properties of the dental materials, which was not possible with traditional *in vitro* assays.

Overall, these studies highlight the potential of microfluidic OoC devices to simulate various microphysiological features of native tissues, and advance our understanding of host–microbe and host–material interactions in dental and periodontal research.

### 4.4. Methods to evaluate outcome of host–microbe interactions using fluidic systems

Evaluating the outcomes of host–microbe interactions using fluidic systems involves a combination of on-chip or off-chip readouts. The optically clear properties of the microfluidic devices, combined with appropriate cellular labeling methods and/or label-free imaging techniques, allow for real-time on-chip visualization and analysis (Fig. 9). Additionally, the fluid flow aspects of the device design enable the continuous collection of cellular secretions over time, providing opportunities for off-chip assessment of the kinetics of biochemical and metabolic secretions. Furthermore, microfluidic devices can be integrated with biosensing modules for real-time assessment of biochemical and biophysical outputs.

Host cells, ECM, and microbes can be visualized on-chip in real-time and following experimental endpoints using fluorescence and confocal microscopy. Live/dead viability assays are commonly employed, with host cells stained with viability dyes such as calcein AM^24^ and bacteria with labels such as SYTO 9 (ref. 26 and 131) and peptidoglycans^24^ to visualize live cells and bacteria in real-time (Fig. 9A and B). Immunostaining on-chip followed by wholemount confocal imaging is also used to visualize and characterize the host cells, ECM, and tissue equivalents. For instance, the cellular organization of fibroblasts within 3D matrices on-chip can be visualized after staining with F-actin^28,31^ and vimentin,^24^ while endothelial cells can be visualized using endothelial markers (CD31, vWF), vascular matrix markers (collagen IV, laminin) and cell junction markers (VE-cadherin, E-cadherin, E-selectin, ICAM-1).^22,28,142^ Similarly, the stratification and differentiation of keratinocytes can be visualized with immunostaining for cytokeratins (CK 5, 10, 14, 13, 19) and barrier proteins (involucrin, filaggrin, loricrin).^27,30,140^ Additionally, label-free imaging modalities such as multiphoton microscopy, second harmonic generation microscopy, and confocal reflectance microscopy are gaining attention for visualizing cellular and extracellular matrix components of cultured tissue equivalents on-chip. Two-photon excited fluorescence from endogenous fluorophores such as nicotinamide adenine dinucleotide, flavin adenine dinucleotide, melanin, and keratins have been utilized for the non-invasive and label-free visualization of keratinocytes, fibroblasts, and endothelial cells.^98,159–161^ Similarly, second harmonic generation microscopy and confocal reflectance microscopy have been used for the label-free visualization of the organization of collagen and other ECM fibers in live tissue constructs.^22,24,98,142,162^ Bacteria in fluidic devices can be visualized using specific stains that label peptidoglycans for their real-time monitoring^24^ or through fluorescence *in situ* hybridization (FISH) as an endpoint assay.^153^ These methods provide insights into the spatiotemporal colonization and invasion of microbes, crucial for understanding oral biofilm formation and interaction with the host tissues.

While imaging-based live/dead assays provide visualization of the live and dead cells, LDH-based assays provide opportunities for timepoint-based kinetics of cytotoxicity assessment without sacrificing the tissue constructs.^24^ The measurement of LDH levels in culture media collected from the outlet ports serves as an off-chip counterpart for the assessment of cell viability and cytotoxicity. The secretion of pro- and anti-inflammatory cytokines and chemokines are key to innate immune response, periodontal pathogenesis, and healing.^163,164^ Enzyme-linked immunosorbent assay (ELISA) is frequently employed to quantify the kinetics of secretion of these soluble factors from the culture media collected from the outlet ports at different time points.^24,28,31,142^ Alternatively, detection and quantification of these soluble factors or inflammatory products could be integrated on-chip through immunosensors incorporated within the chip or as an independent biosensing module. Previous studies have demonstrated the incorporation of biosensors for the detection and quantification of proteins like interleukin-6, interferon-γ, tumor necrosis factor-α, and nitric oxide.^165–167^ Similarly, OoC platforms where oxygen gradient maintenance is crucial to recapitulate the anaerobic environment of periodontal pathogens, integration of oxygen sensing probes can provide real-time, non-invasive monitoring of the gaseous microenvironmental parameters and oxygen gradient within the chip.^115,168^

Computational fluid dynamics is frequently used to understand the impact of fluid flow dynamics within the microfluidic chip and to estimate the shear stresses experienced by host cells and bacteria, as well as the diffusion of macromolecules.^24,153^ This can be correlated with on-chip assays like diffusion assays and FRAP to measure diffusion kinetics of macromolecules of varying molecular weight.^24,28^ These diffusion assays can also be used to assess the integrity of barrier tissues including epithelial and endothelial layers. The impact of periodontal microbes on the barrier properties of the tissues can be further quantified using TEER.^31^ Electrophysical sensors like TEER probes can also be integrated on-chip which provides real-time, label-free monitoring of the ohmic resistance or impedance that can be translated to the measurement of tight junction integrity of epithelial barriers in OoC devices.^168,169^

Overall, these methods offer a comprehensive toolkit to evaluate the multifaceted outcomes of host–microbe interactions and real-time monitoring of complex biological processes. Moving forward, the integration of multi-omics technologies and advanced imaging techniques with fluidic systems holds promise for uncovering novel insights into host–microbiome interactions.

## 5. Future directions and conclusions

### 5.1. Insights and inspirations from gut-on-chip systems

The oral and gut environments, although distinct in their primary functions and anatomical locations, exhibit several key similarities, particularly in terms of their complex microbiota, immune interactions, and the presence of anaerobic–aerobic interfaces. Both the oral cavity and the gut host diverse microbial communities that play essential roles in maintaining host physiological homeostasis. Play crucial roles in educating the host immune system, aiding in nutrient digestion, and providing defense against colonization by pathogenic microorganisms.^170^ Although the mucosal tissues of the oral and gut environments differ in tissue-level architecture and specific functions, both are highly vascularized and contain extensive networks of immune cells that contribute to immune surveillance and the regulation of immunological homeostasis. Additionally, the fluid flow properties within these environments (saliva in the oral cavity and mucus in the gut) serve to protect and maintain epithelial integrity by facilitating the removal of debris and pathogens. Emerging evidence supports the concept of a gut-oral axis, wherein bidirectional interactions between these compartments influence microbial composition and systemic homeostasis, highlighting their interconnected nature.^170–172^ These similarities underscore the potential for translating insights from gut-on-chip models to periodontal research, offering valuable perspectives on host–microbiome interactions and systemic influences.

**Fig. 10:**
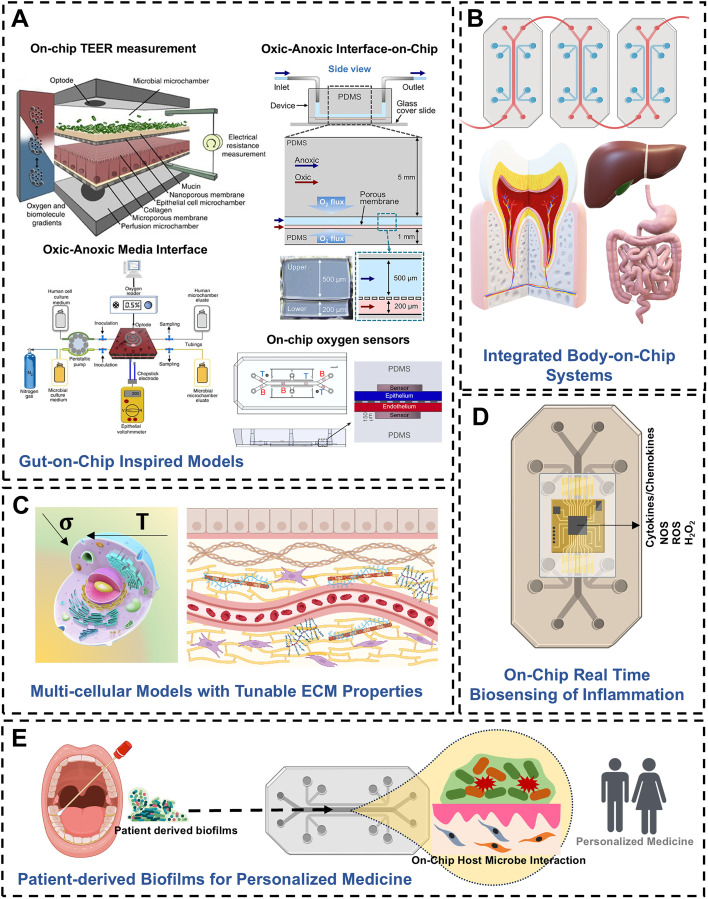
Future directions in the development of OoC systems to study periodontal host–microbe interactions. (A) Gut-on-chip models incorporating TEER electrodes for assessing epithelial barrier integrity, establishment of oxic-anoxic interfaces and integration of oxygen sensors for culture and monitoring of anaerobic microbes with host cells. (B) Integrated body-on-chip (BoC) systems to simulate periodontal and systemic interactions, incorporating components of the oral cavity and distant organs like the gut and liver. (C) ECM induced mechanical cues such as shear stress, tension, and compression experienced by host cells and how future OoC models can enable culture of cells in artificial ECMs with tunable mechanical properties. (D) Integration of biosensors in OoC systems for real-time biosensing of mediators of periodontal inflammation. (E) Culture of patient-derived biofilms on-chip to enhance disease modeling, drug discovery and screening, personalized dental medicine. Figure panel A is adapted from ref. 168 under the terms of the CC-BY license, ref. 114 under the terms of the CC-BY license, and ref. 115 with permission from Springer Nature Ltd., ©2019. Figure panels B–E created with https://Biorendor.com.

Gut-on-chip models have advanced significantly, overcoming limitations of traditional *in vitro* systems, and these advancements can be adapted for periodontal research to provide more physiologically relevant models (Fig. 10). For instance, mechanically-active gut-on-chip models have successfully recreated the dynamic environment of the gut, including peristalsis-like motion and continuous fluid flow.^173–175^ These systems typically use PDMS-based devices with two channels separated by a porous membrane, allowing nutrient diffusion and epithelial cell attachment. The incorporation of mechanical deformation through controlled vacuum or air pressure allows rhythmic mechanical deformation of the epithelial cell monolayer, resembling peristalsis-like motion. Studies have demonstrated that continuous perfusion and incorporation of peristalsis-like motion leads to enhanced epithelial differentiation, villus formation, and barrier function of the intestinal epithelium.^122,170,173–177^ Similar devices could be adapted to replicate the mechanical forces found in the oral cavity, such as chewing, orthodontic movement, salivary flow, and brushing, to study their impact on the oral epithelium and microbial interactions in periodontal disease studies.

Gut-on-chip systems have effectively incorporated oxic–anoxic interfaces, which are crucial for maintaining host cell function and supporting the growth of commensal anaerobes^178^ (Fig. 10A). This has been facilitated by adjusting the thickness of PDMS slabs and employing gas-impermeable materials such as polycarbonate, and designing dedicated inlets and outlets for oxic media (21% dissolved oxygen) for host cell culture and anoxic media (0.1% dissolved oxygen) for microbial culture.^168^ Similar design features could be adapted to recapitulate the anoxic microenvironment of periodontal pockets, thereby enabling the study of interactions between periodontal tissues and complex biofilms containing anaerobic periodontal pathogens. Alternatively, existing periodontal OoC systems could be incorporated with oxygen scavengers such as oxyrase, sodium sulfite to generate oxygen gradients.^179^ Gut-on-chip platforms have demonstrated the integration of biosensors for real-time assessment of biochemical and biophysical outputs, such as oxygen levels and barrier integrity^114,115,168^ (Fig. 10A). Advancements in biosensing technologies and its cross application^180^ in the context of oral microenvironment that includes sensitivity and specificity optimization under salivary pH conditions, can enable real-time monitoring of key parameters like inflammatory cytokine levels, tissue permeability, and microbial metabolite production, thereby providing a comprehensive understanding of disease progression and host responses.

The application of gut-on-chip systems has also expanded to model inflammatory gut diseases *in vitro*.^175,181^ Features of enteric pathologies ranging from degradation of epithelial barrier permeability,^175,181^ induction of cytopathic effect,^182^ and production of pro-inflammatory cytokines^115,175,181^ after challenging the intestinal equivalents on-chip with entero-invasive bacteria have been demonstrated. These studies have also incorporated and assessed the role of immune cells like peripheral blood mononuclear cells^175^ and macrophages^181^ towards barrier function and host immune polarization with and without the presence of enteropathogens. This progress has already inspired the development of analogous oral disease models. For instance, Svanberg *et al.*^142^ developed vascularized periodontal tissues within a microfluidic chip and demonstrated the formation of perfusable vascular networks, with periodontal ligament cells enhancing blood vessel formation and maturation. The study also showed significant upregulation of inflammatory cytokines such as IL-6, IL-8, and IL-18 in response to LPS from *E. coli*, highlighting its relevance for studying early stages of periodontitis. Similarly, using vascularized gingival tissue equivalents, Makkar *et al.*,^22^ demonstrated the potential to study vascular and immune cell polarization events in periodontal disease using co-culture with an array of early, intermediate, and late biofilm colonizers. The incorporation of vasculature provided opportunities to visualize the intra-vascular invasion of pathogenic bacteria, potentially paving the way towards studying oral-systemic links of periodontal disease. Furthermore, the secreted factors from the vascularized tissue models that were exposed to these bacterial colonizers showed distinct polarization of macrophages into either pro-inflammatory or anti-inflammatory states, providing insights into host tissue activation and tissue destruction. Future studies incorporating the insights from advanced gut-on-chip models into gingival and periodontal tissues on-chip models can provide deeper insights into epithelial barrier disruption, bacterial invasion, inflammatory response, and oral-systemic influences of periodontal disease.

Overall, the advancements in gut-on-chip platforms provide a robust framework for developing similar systems to study host–microbe interactions in periodontal disease. By leveraging these insights, periodontal disease models can achieve greater physiological relevance, enhancing our understanding of disease mechanisms and paving the way for novel periodontal therapeutic strategies.

### 5.2. Recapitulating the diverse oral microbiome and exploring inflammatory comorbidities

The oral microbiome is composed of diverse microbial species that interact intricately with each other and the host. Future models that replicate these complex microbial communities, composing both commensal and pathogenic species, will offer a more accurate representation of the oral ecosystem and its impact on periodontal health and disease. Developing platforms with long-term host–microbe co-culture capabilities will facilitate the recapitulation of complex microbial communities, including the transition from symbiotic to dysbiotic biofilms, and their influence on periodontal disease initiation and progression.

Epidemiological, clinical interventional, and experimental studies collectively provide strong evidence that periodontitis adversely affects overall health.^117,118^ Furthermore, targeted therapies aimed at specific periodontal pathogens, such as *P. gingivalis* and *F. nucleatum*, could potentially reduce the severity of associated comorbidities. Future work should focus on elucidating the mechanisms linking periodontitis to these comorbidities through bioengineered multi-tissue models. Evidence suggests that inflammation-adapted hematopoietic progenitors might serve as a bridge between periodontitis and cardiovascular disease aligns, aligning with clinical imaging data that show a correlation between periodontal inflammation and both hematopoietic tissue activity and arterial inflammation.^119^ Additionally, emerging evidence suggests that inflammatory processes in peripheral tissues such as periodontal tissues may be interconnected through adaptations in the bone marrow, which acts as inductive site.^118^ Understanding these connections could provide new insights into the mechanisms underlying comorbidities and shift current perspectives on inflammation and disease. The concept of inflammatory adaptation in hematopoietic progenitors could provide a unifying framework linking peripheral and central inflammation, presenting a potential platform for innovative therapeutic approaches targeting inflammation and related comorbidities. However, this concept requires further exploration in future research. As OoC technology continue to advance, integrating multiple organ systems-on-chip (Fig. 10B), can be instrumental in elucidating the complex interactions between oral and systemic conditions such as cardiovascular disease, inflammatory bowel disease, and diabetes. Overall, these advancements promise to deeper understanding of periodontal disease mechanisms, oral-systemic influences, and the development of more effective diagnostic, preventive, and therapeutic strategies.

### 5.3. Recapitulation of cellular, matrix, and immune interactions

3D organotypic models and organ-on-chip systems of gingival and periodontal tissues have provided opportunities to recapitulate various key features of the native tissue microenvironment, its interaction with microbes, and its influence on periodontal health and disease. Key areas for advancement include increasing the cellular and architectural complexity of the gingival epithelium and connective tissue microenvironment. The inclusion of immune and vascular components remains a critical area to explore. Integrating immune cells, such as macrophages and neutrophils, into these models can offer a more comprehensive understanding of the immune response to periodontal microbiota and pathogens. Additionally, incorporating microvascular networks will allow for the study of nutrient exchange, immune cell trafficking, systemic dissemination of bacterial components, and their systemic influences.

The ECM of gingival tissues exhibits a distinct composition and structure that varies between health and disease states. In periodontal disease, the ECM undergoes significant degradation due to an imbalance between ECM synthesis and remodeling processes.^2,39,183^ Beyond providing structural support, the ECM plays a crucial role in modulating host immune responses. Upon contact with the ECM, immune cells receive essential cues for survival, proliferation, differentiation, and activation, as well as support for adhesion and guidance for migration. This intricate interplay underscores the importance of ECM–immune system interactions, where ECM-derived signals coordinate immune responses and immune cells, in turn, facilitate ECM repair and regeneration.^184^ Recapitulating the mechanical properties of gingival connective tissue through organotypic culture and OoC approaches will allow for a deeper mechanobiological understanding of periodontal inflammation. Biomaterials with tunable mechanical properties^185^ can be employed as artificial ECMs for both organotypic and on-chip cultures, enabling the investigation of mechanical regulation of inflammation in these tissues (Fig. 10C).

### 5.4. On-chip biosensing and personalized dental medicine

The future development of organ-on-chip systems, enhanced with integrated biosensors, holds significant promise for advancing our understanding of periodontal host–microbe interactions (Fig. 10D). As these technologies evolve, they will facilitate real-time, dynamic monitoring of key host and microbial markers that orchestrate immune responses during the phases of infection and inflammation.^186^ Biosensors with enhanced sensitivity and specificity will enable the detection of subtle changes in immune signaling, providing insights into the early stages of infection, microbial persistence, and host immune modulation.

Furthermore, as these biosensor-enabled platforms become more sophisticated, they can play a transformative role in personalized medicine (Fig. 10E). By allowing for the monitoring of individual immune responses and microbial interactions within a controlled, human-relevant environment, OoC systems could be employed to assess drug efficacy, predict patient-specific responses to treatments, and optimize therapeutic regimens for infectious diseases and inflammatory conditions. Ultimately, these advances could expand the horizons of drug discovery, precision medicine, and disease modeling, offering more accurate and predictive tools for both basic research and clinical applications.^122,125,126^

## Conflicts of interest

There are no conflicts of interest to declare.

## Data Availability

No primary research results, software or code have been included and no new data were generated or analysed as part of this review.
